# Supplementary material for: Lycorine Carbamate Derivatives for Reversing P-glycoprotein-Mediated Multidrug Resistance in Human Colon Adenocarcinoma Cells
Source: Int J Mol Sci. 2023 Jan 20;24(3):2061. doi: 10.3390/ijms24032061 (PMC9916770; doi:10.3390/ijms24032061)
Supplement: Supplementary file 1 [file ijms-24-02061-s001.zip › ijms-2078109-supplementary.pdf]

Supporting information

Lycorine carbamate derivatives for reversing P-glycoprotein mediated multidrug resistance in human colon adenocarcinoma cancer cells.

Shirley A. R. Sancha [1], Nikoletta Szemerédi[2], Gabriella Spengler[2], and Maria-José U. Ferreira[1*]

[1] Research Institute for Medicines (iMed.ULisboa), Faculty of Pharmacy, Universidade de Lisboa, Av. Prof. Gama Pinto, 1649-003 Lisbon, Portugal.

[2] Department of Medical Microbiology, Albert Szent-Györgyi Health Center, Faculty of Medicine, University of Szeged, Semmelweis utca 6, 6725 Szeged, Hungary

***Corresponding author:** Maria-José U. Ferreira; Email: mjuferreira@ff.ulisboa.pt.

Table of contents

1. **NMR data of parental compound 1** S3
2. **Representative ^{1}H and ^{13}C NMR spectra** S4
3. **Rhodamine-123 accumulation assay (compounds 1 – 32)** S19
4. **Flow cytometry data** S21
6. **Combination chemotherapy results** S26
7. **Physicochemical properties** S27
8. **Pharmacokinetic properties** S28
9. **References** S29

1. NMR data of parental compound 1

Lycorine (1)

Amorphous powder. $[\alpha]_D^{25} - 70.6$ (*c* 0.31, MeOH); IR ν_{max} cm^{-1} (KBr): 3334, 1485, 744. ESI-MS (positive mode) m/z (rel. int) 288 $[M + H]^+$ (100). ^1H NMR (300 MHz, DMSO-d_6) δ 6.80 (1H, *s*, H-10), 6.67 (1H, *s*, H-7), 5.95 (2H, *dd*, *J* = 4.1, 0.9 Hz,O<u>CH₂</u>O), 5.37 (1H, *bs*, H-3), 4.85 (1H, *d*, *J* = 6.2 Hz, 2-OH), 4.75 (1H, *d*, *J* = 4.2 Hz, 1-OH), 4.27 (1H *bs*, H-1), 4.04 (1H, *d*, *J* = 14.2 Hz, H-6β), 3.96 (1H, *bs*, H-2), 3.29 (1H, *d*, *J* = 14.2 Hz, H-6α), 3.18 (1H, *ddd*, *J* = 9.1, 7.2, 2.1 Hz, H12-β), 2.61 (1H, *d*, *J* = 10.4 Hz, H-10b), 2.51 (1H, *m*, H-4a), 2.47 (2H, *m*, H-11), 2.20 (1H, *q*, *J* = 8.5 Hz, H-12α) ppm. ^{13}C NMR (75 MHz, DMSO- d_6) δ 145.6 (C-9), 145.1(C-8), 141.6 (C-4), 129.7(C-6a), 129.5(C-10a), 118.4 (C-3), 106.9 (C-7), 105.0 (C-10), 100.5 (O<u>CH₂</u>O), 71.7 (C-2), 70.2 (C-1), 60.7 (C-4a), 56.7 (C-6), 53.2 (C-12), 40.1(C-10b), 28.1 (C-11) ppm.

2. Representative ^{1}H and ^{13}C NMR spectra

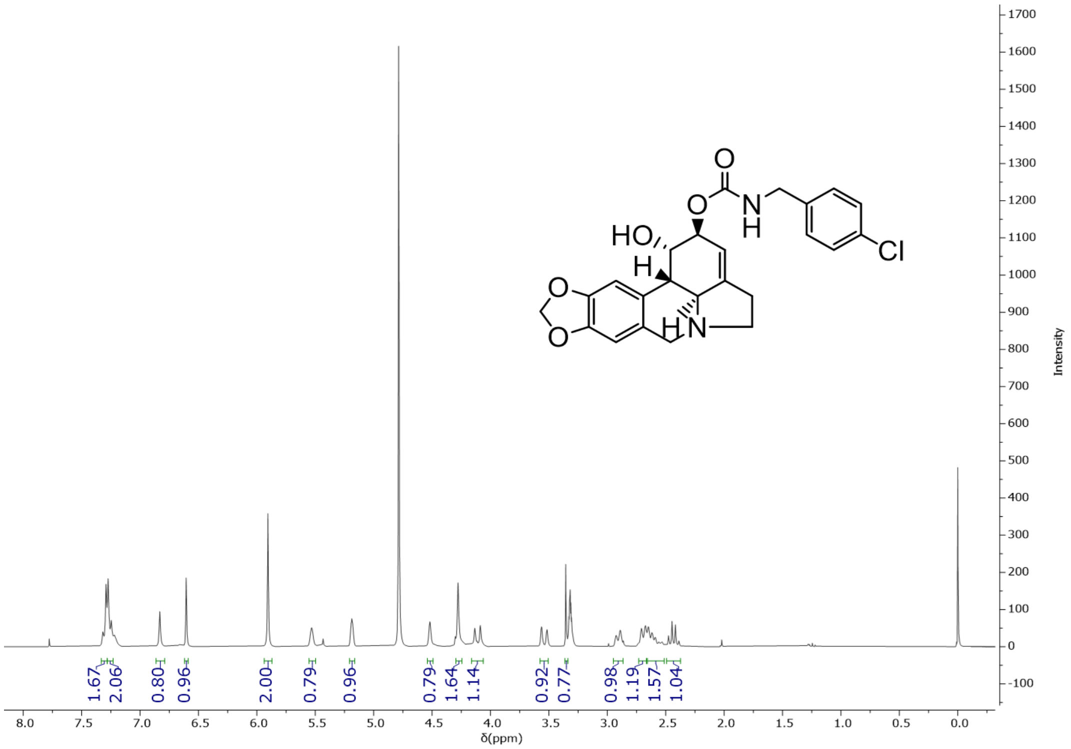

Figure S1: ^{1}H-NMR spectrum of compound **2** (300 MHz, $CDCl_3/CD_3OD$).

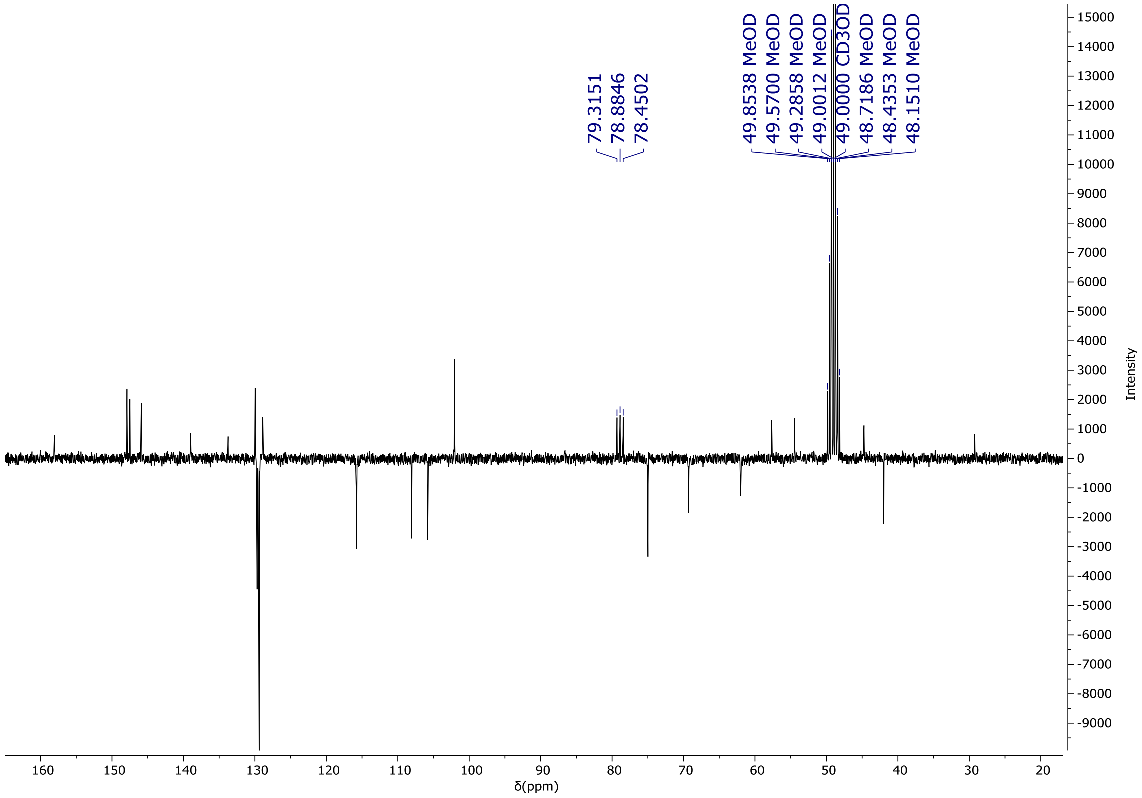

Figure S2: ^{13}C-APT NMR spectrum of compound **2** (75 MHz, $CDCl_3/CD_3OD$).

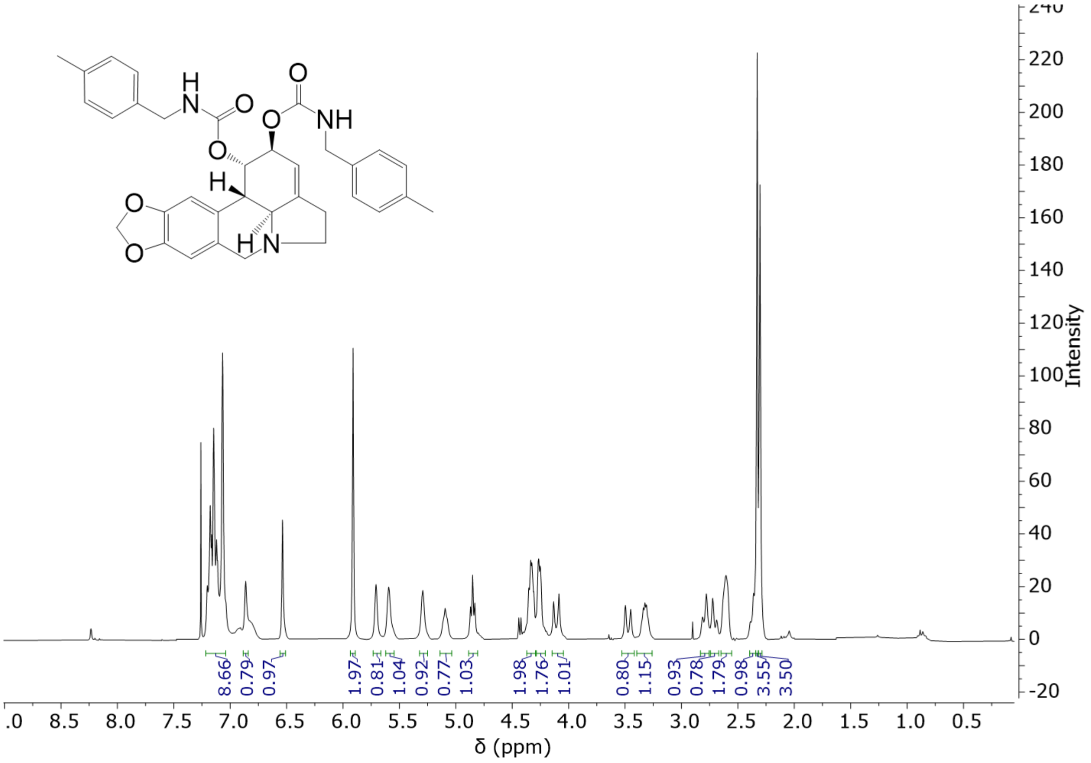

Figure S3: ^{1}H-NMR spectrum of compound **5** (300 MHz, $CDCl_3$).

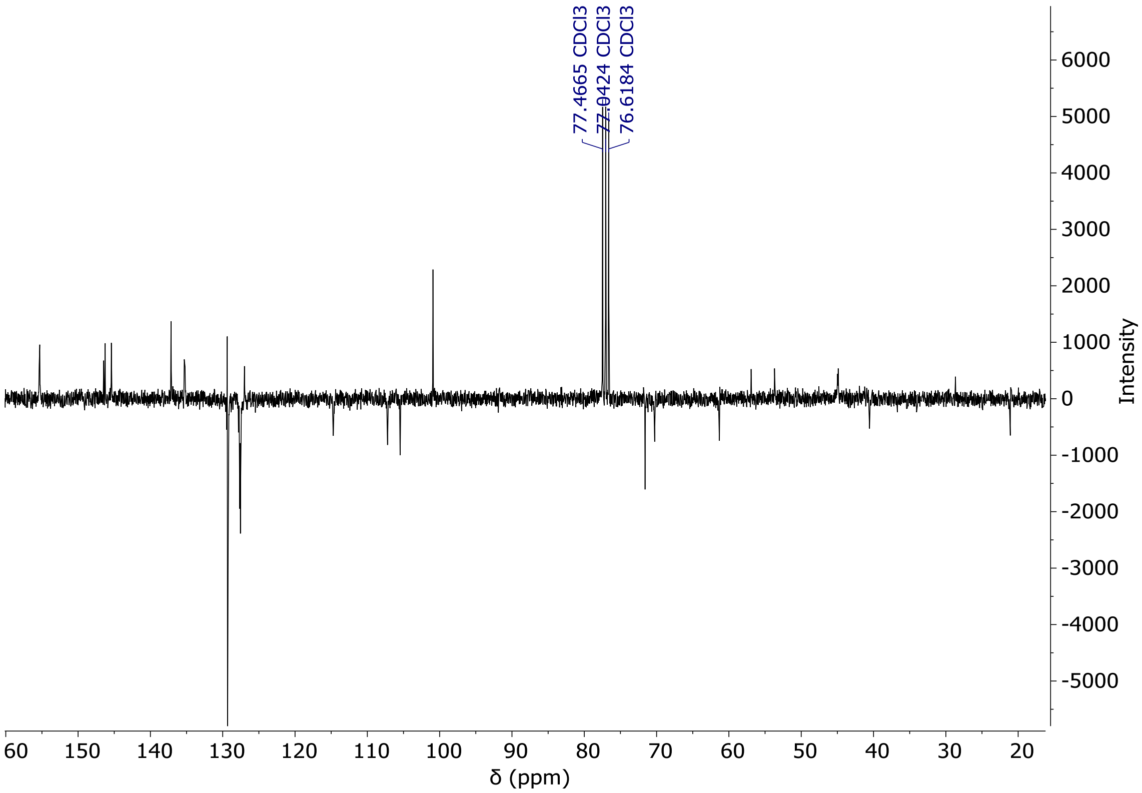

Figure S4: ^{13}C-APT NMR spectrum of compound **5** (75 MHz, $CDCl_3$).

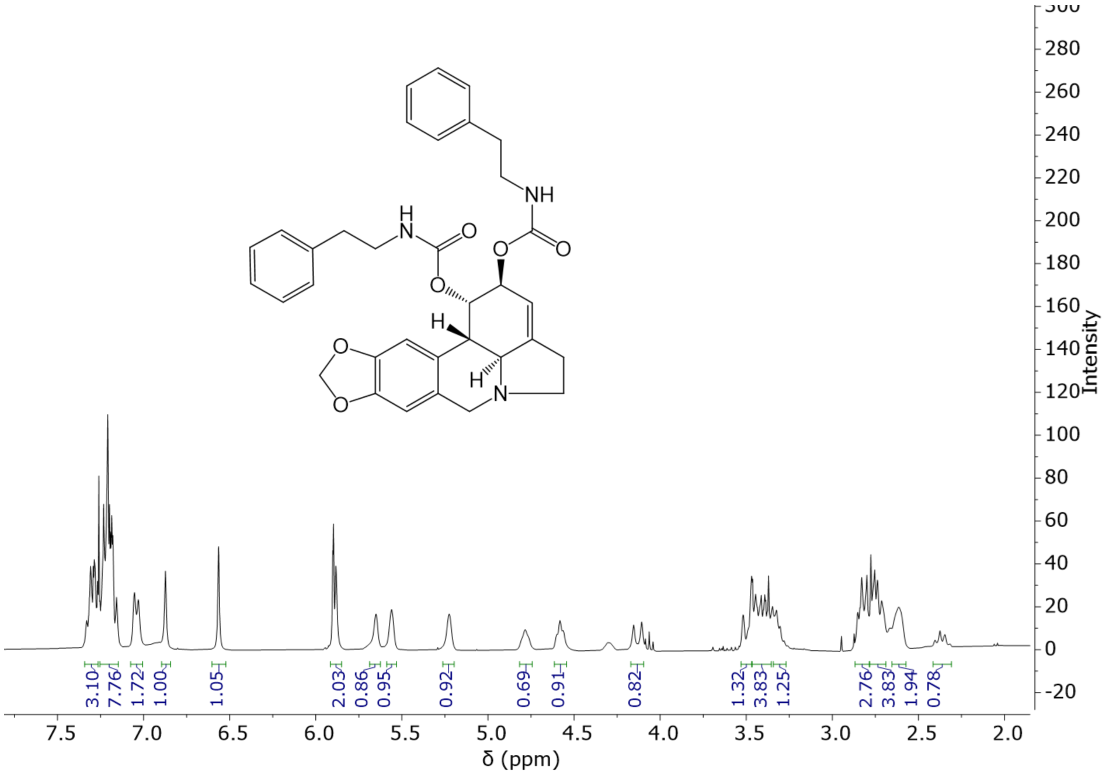

Figure S5: ^{1}H-NMR spectrum of compound **9** (300 MHz, $CDCl_3$).

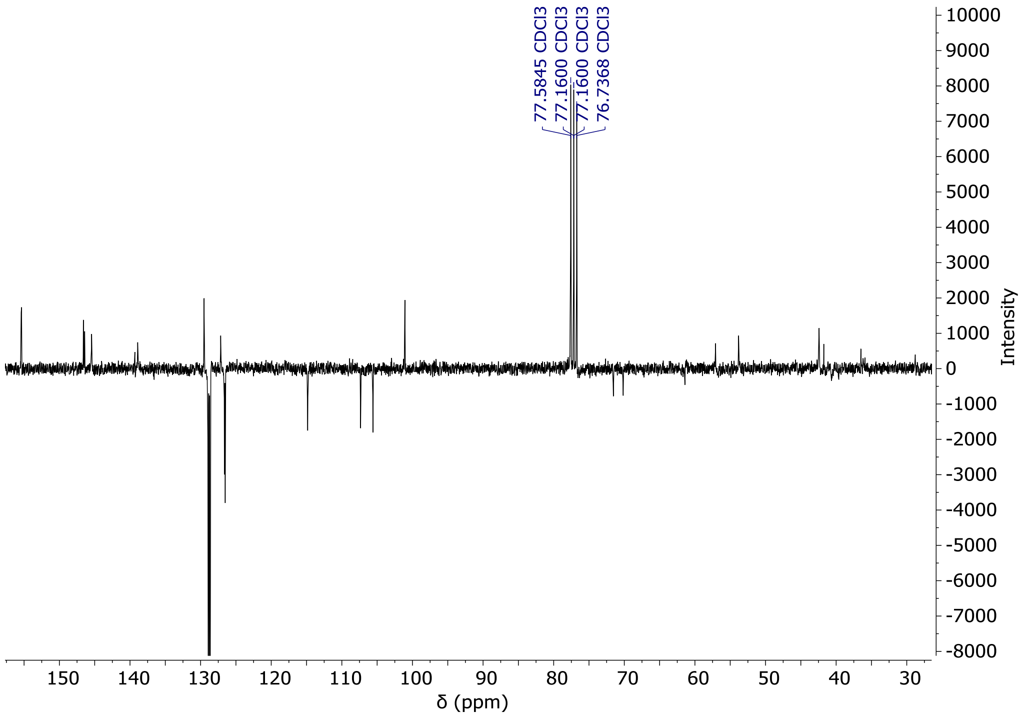

Figure S6: ^{13}C-APT NMR spectrum of compound **9** (75 MHz, $CDCl_3$).

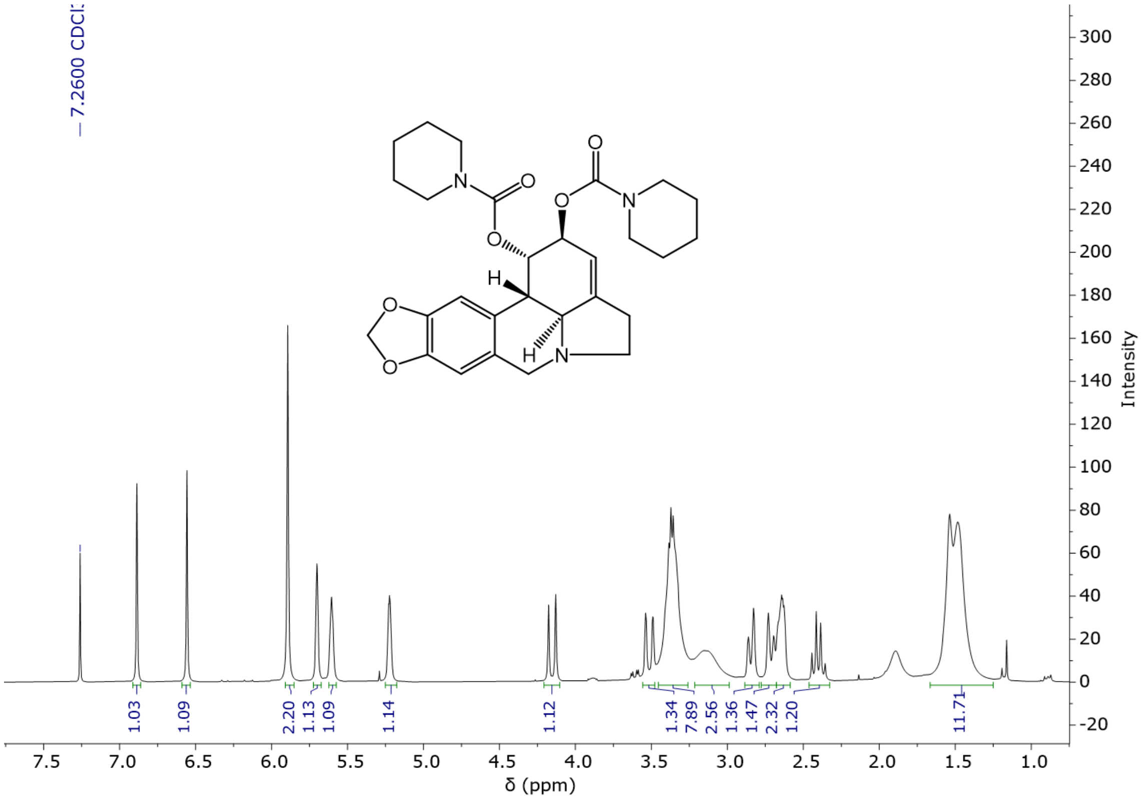

Figure S7: ^{1}H-NMR spectrum of compound **16** (300 MHz, $CDCl_3$).

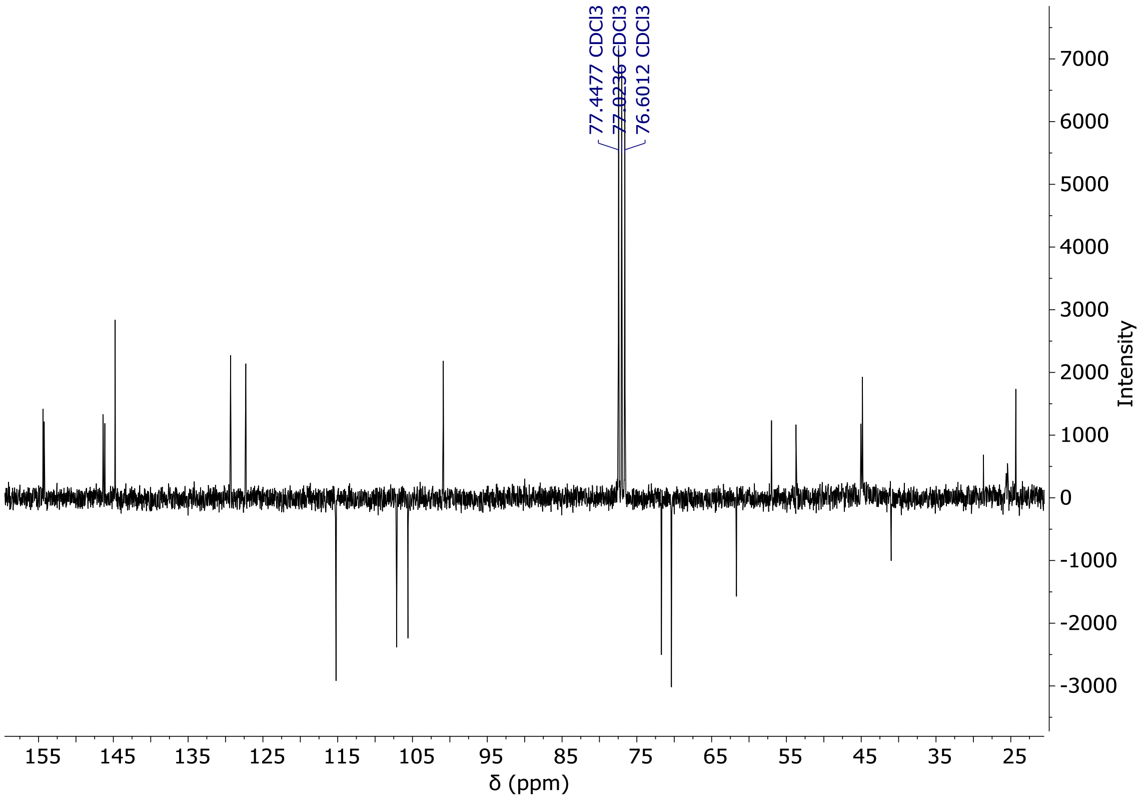

Figure S8: ^{13}**C**-APT NMR spectrum of compound **16** (75 MHz, $CDCl_3$).

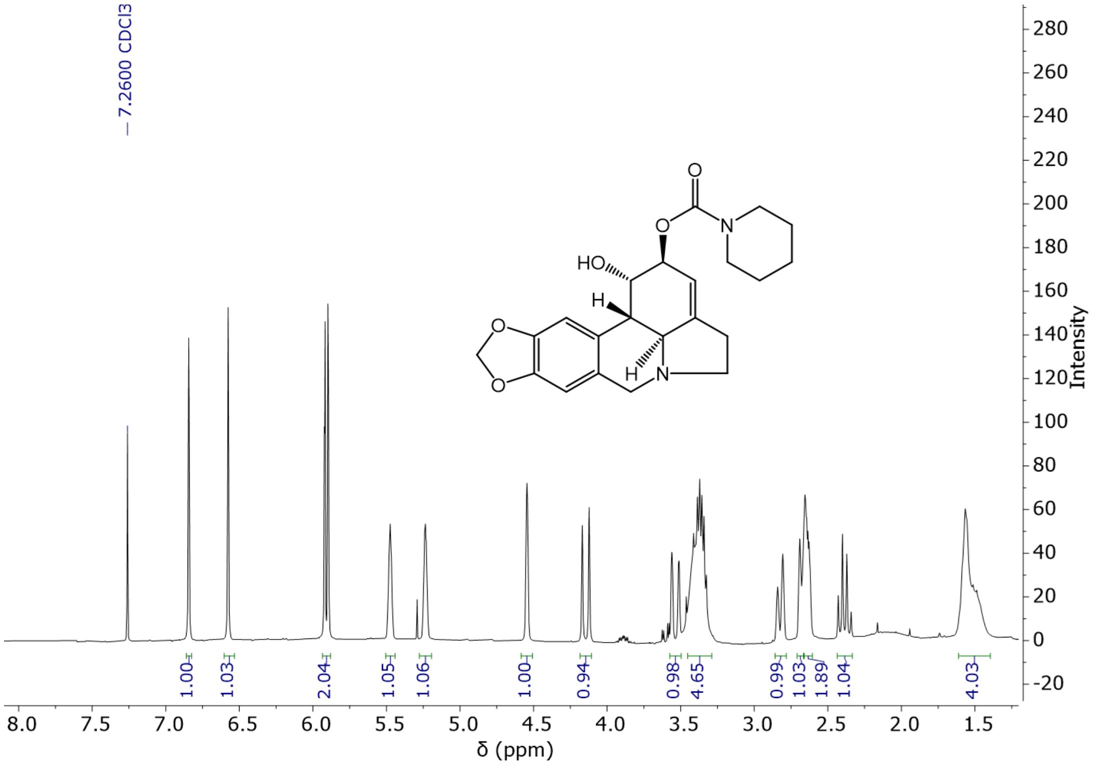

Figure S9: ^{1}H-NMR spectrum of compound **17** (300 MHz, $CDCl_3$).

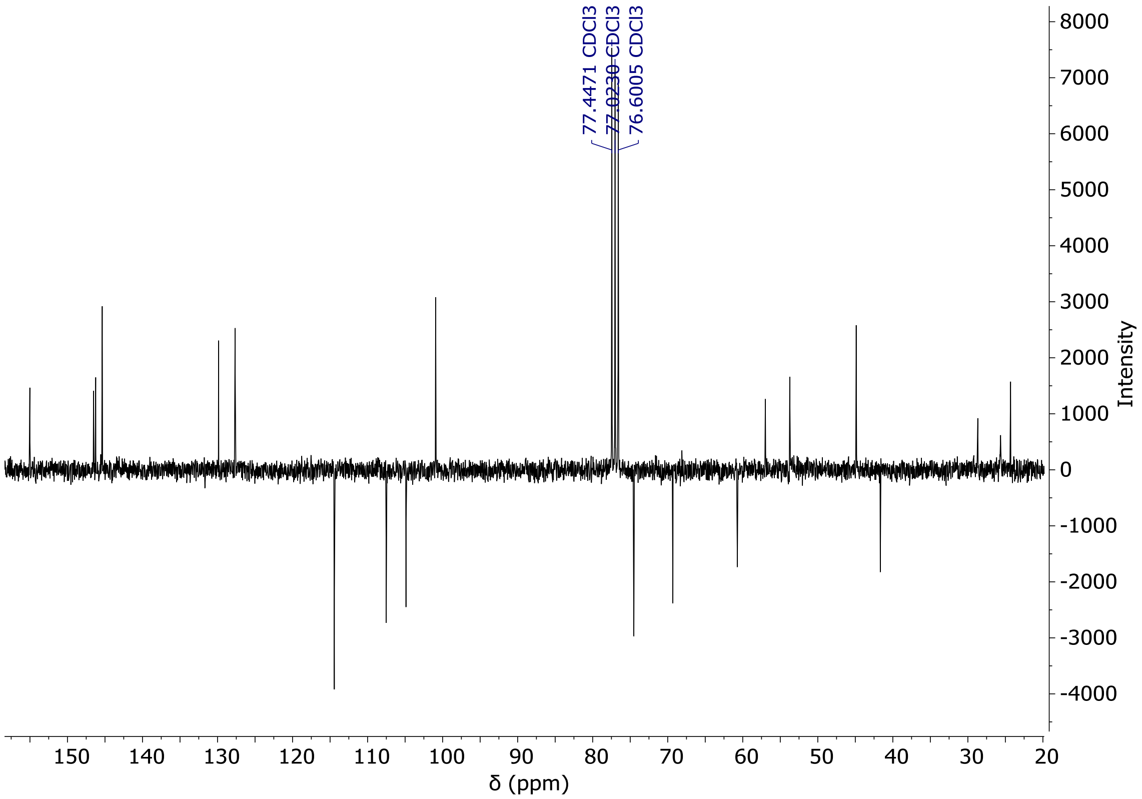

Figure S10: ^{13}C-APT NMR spectrum of compound **17** (75 MHz, $CDCl_3$).

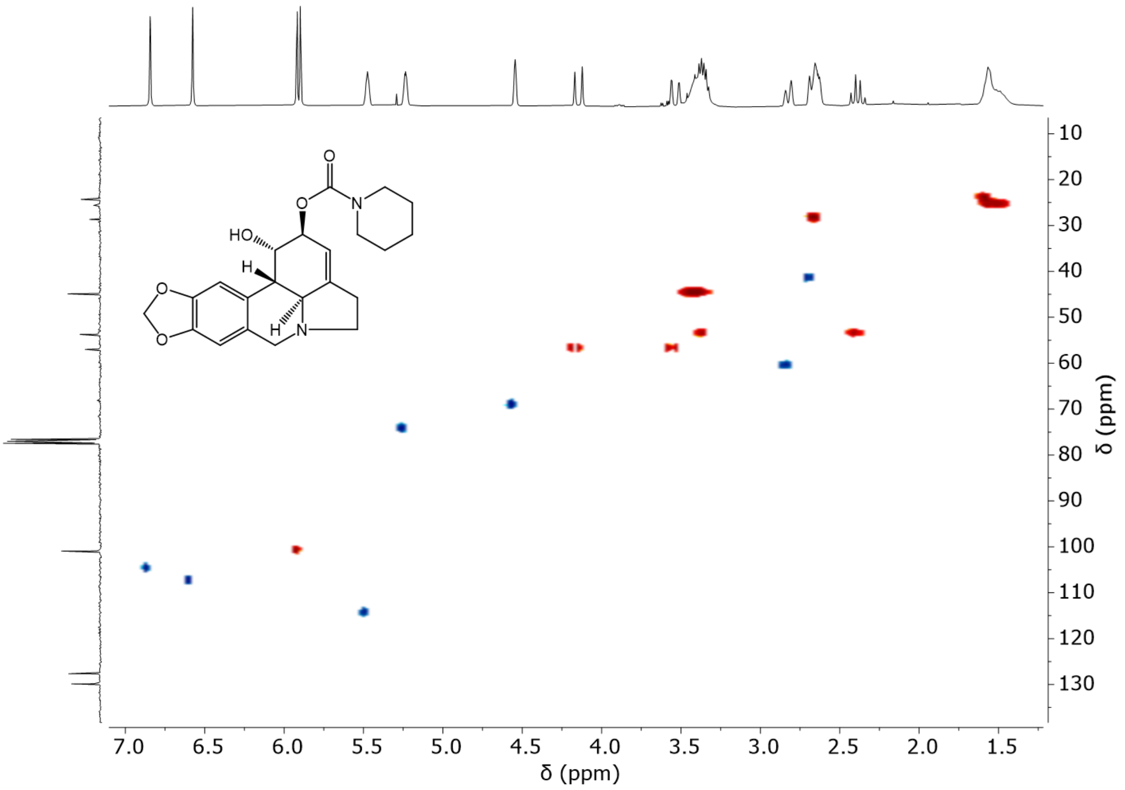

Figure S11: HSQC spectrum of compound **17.**

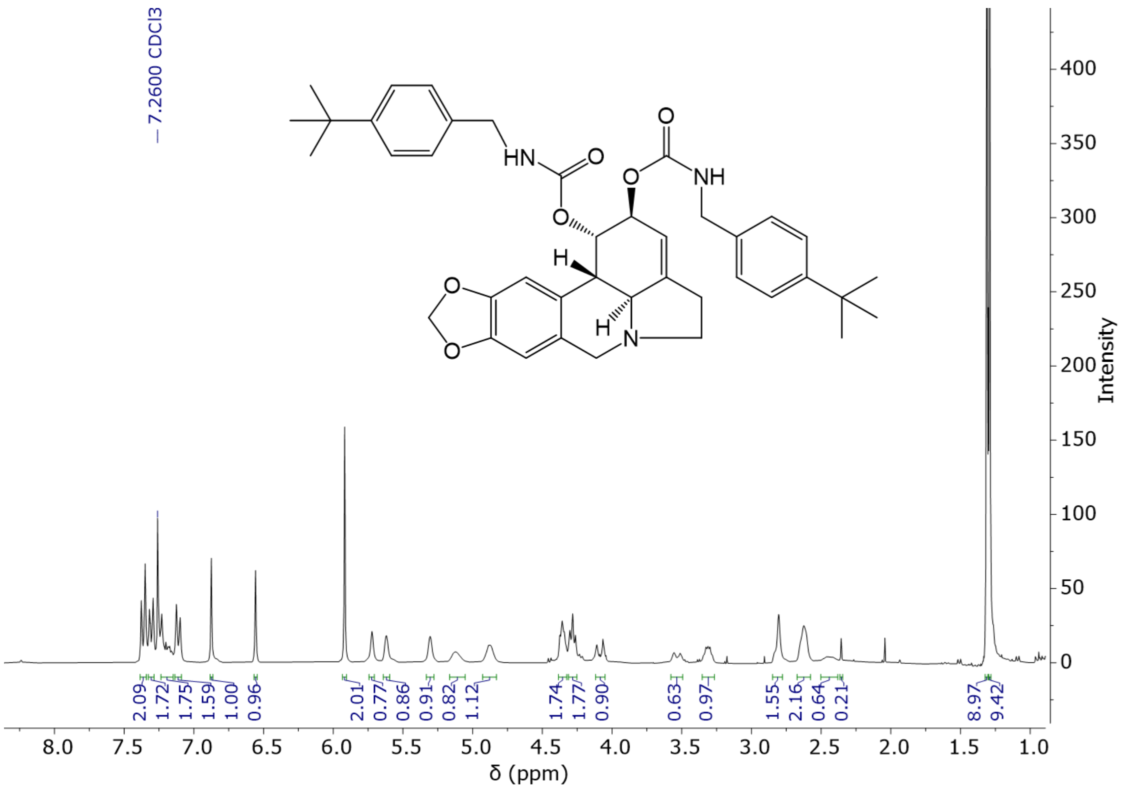

Figure S12: ^{1}H-NMR spectrum of compound **18** (300 MHz, $CDCl_3$).

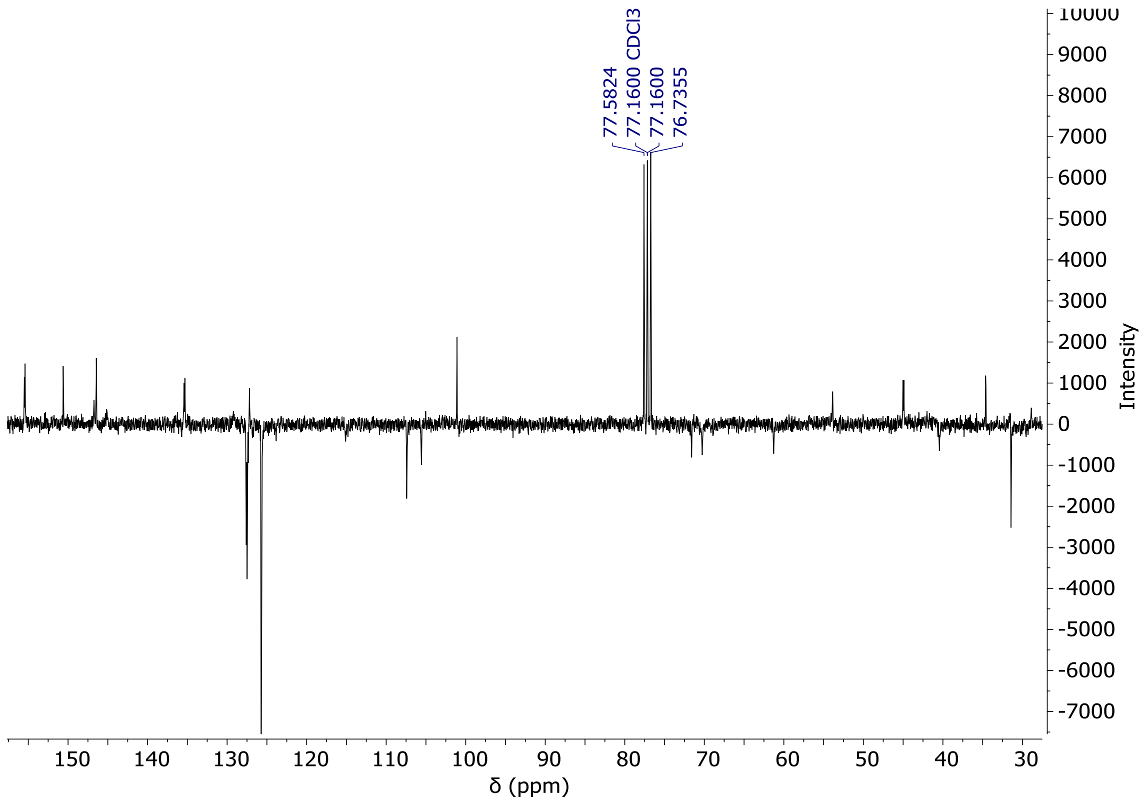

Figure S13: ^{13}C-APT NMR spectrum of compound **18** (75 MHz, $CDCl_3$).

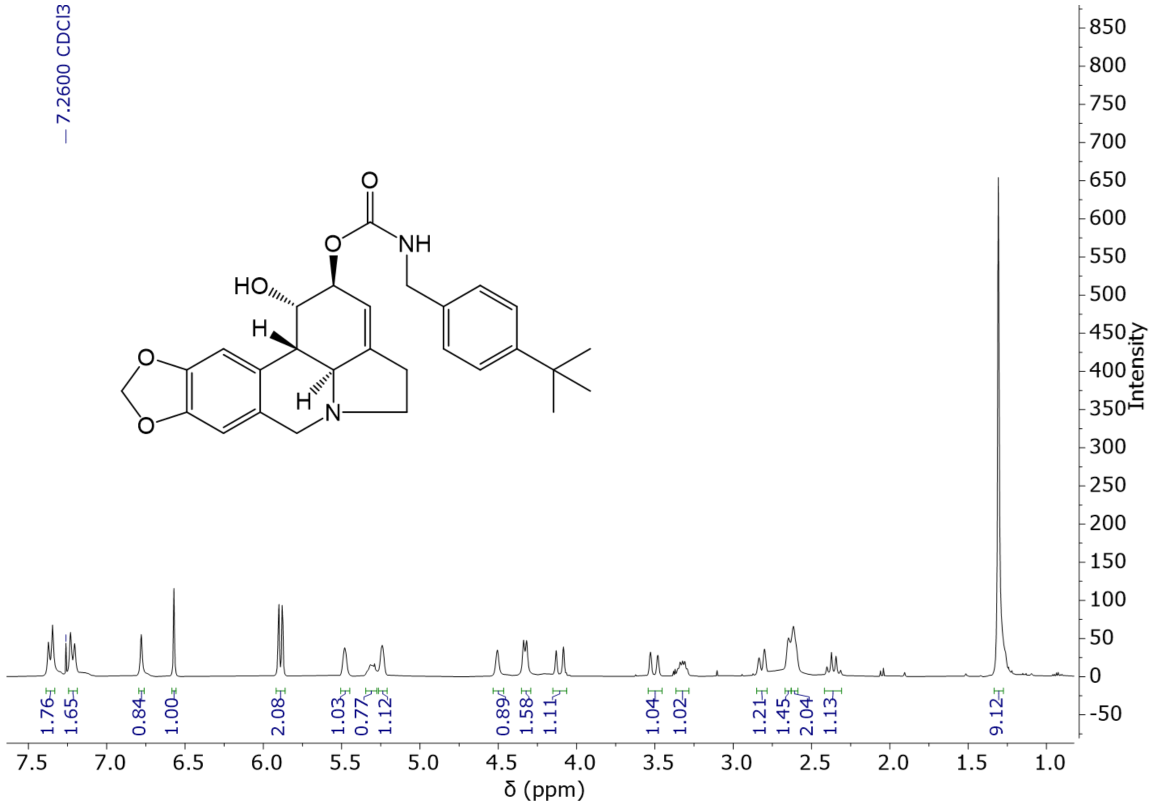

Figure S14: ^{1}H-NMR spectrum of compound **19** (300 MHz, $CDCl_3$).

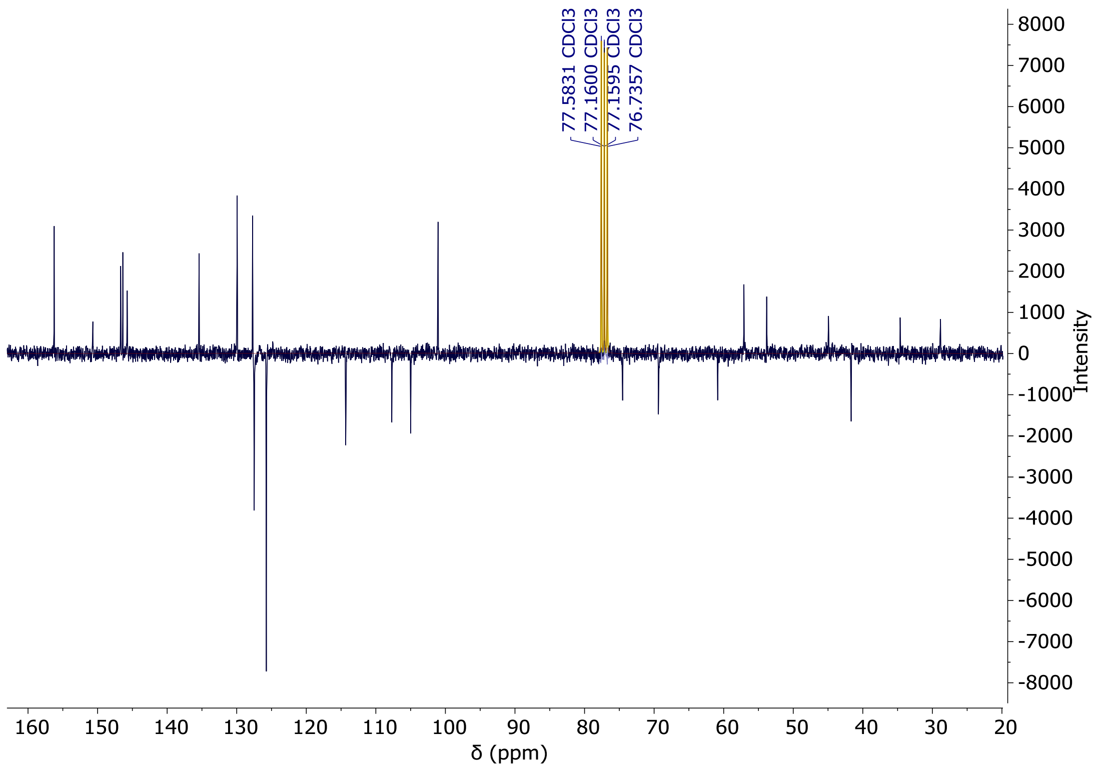

Figure S15: ^{13}C-APT NMR spectrum of compound **19** (75 MHz, $CDCl_3$).

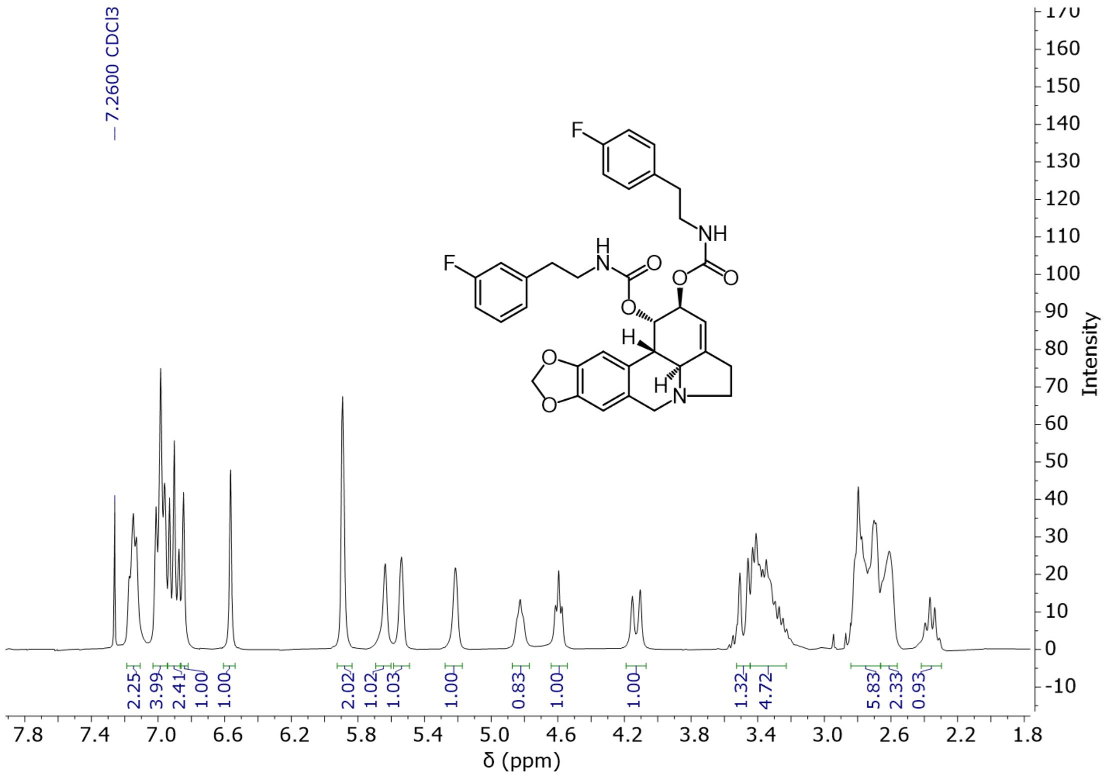

Figure S16: ^{1}H-NMR spectrum of compound **23** (300 MHz, $CDCl_3$).

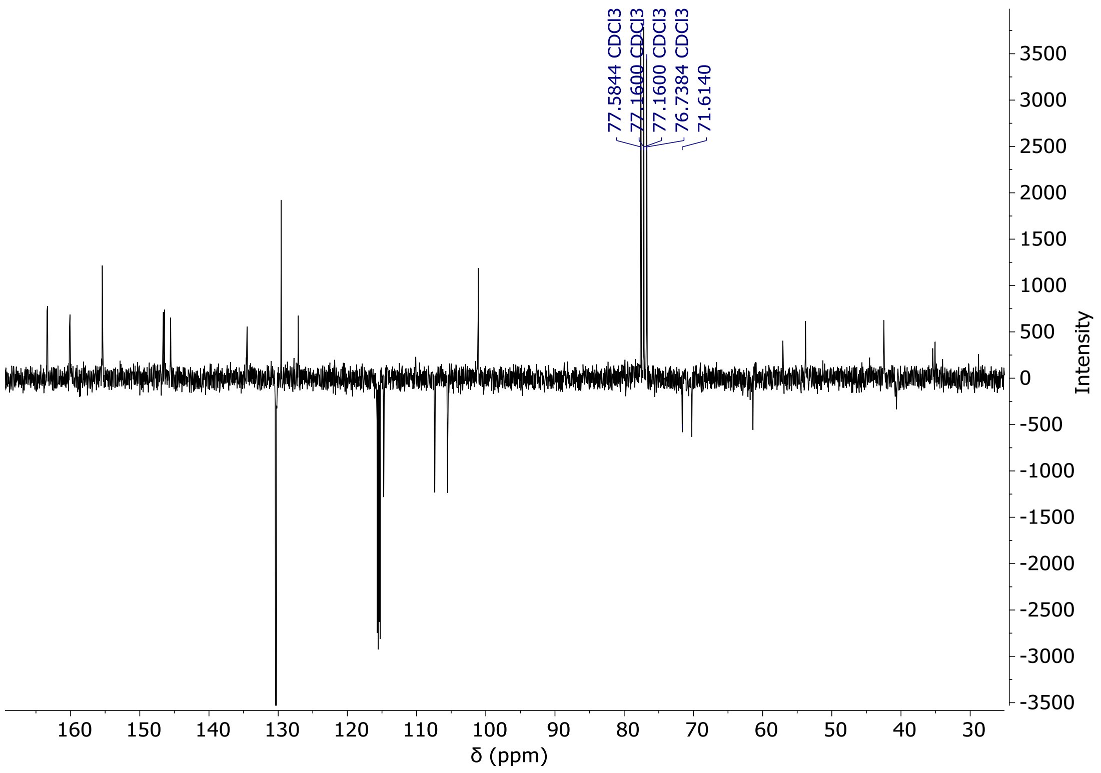

Figure S17: ^{13}C-APT NMR spectrum of compound **23** (75 MHz, $CDCl_3$).

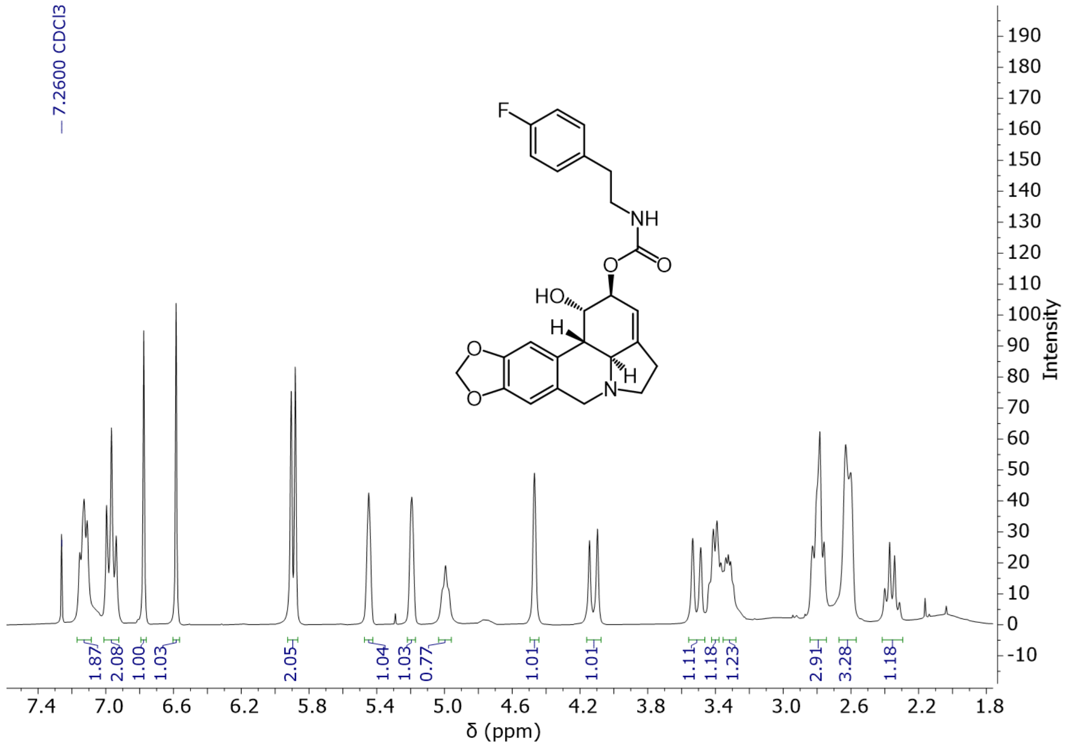

Figure S18: ^{1}H-NMR spectrum of compound **24** (300 MHz, $CDCl_3$).

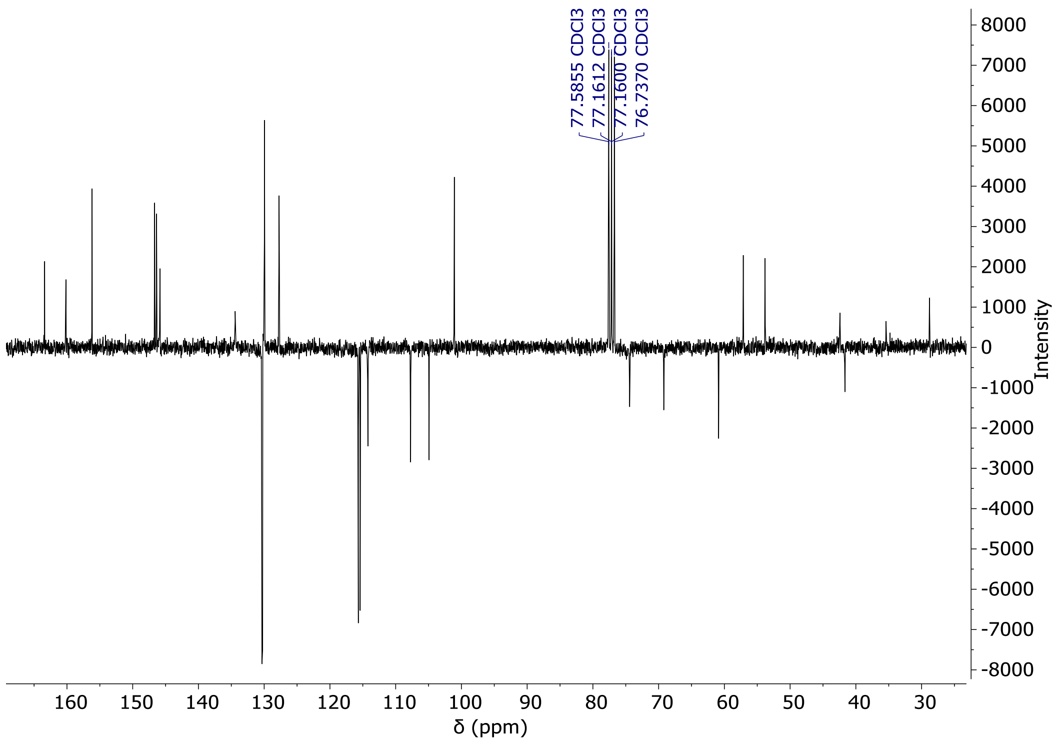

Figure S19: ^{13}C-APT NMR spectrum of compound **24** (75 MHz, $CDCl_3$).

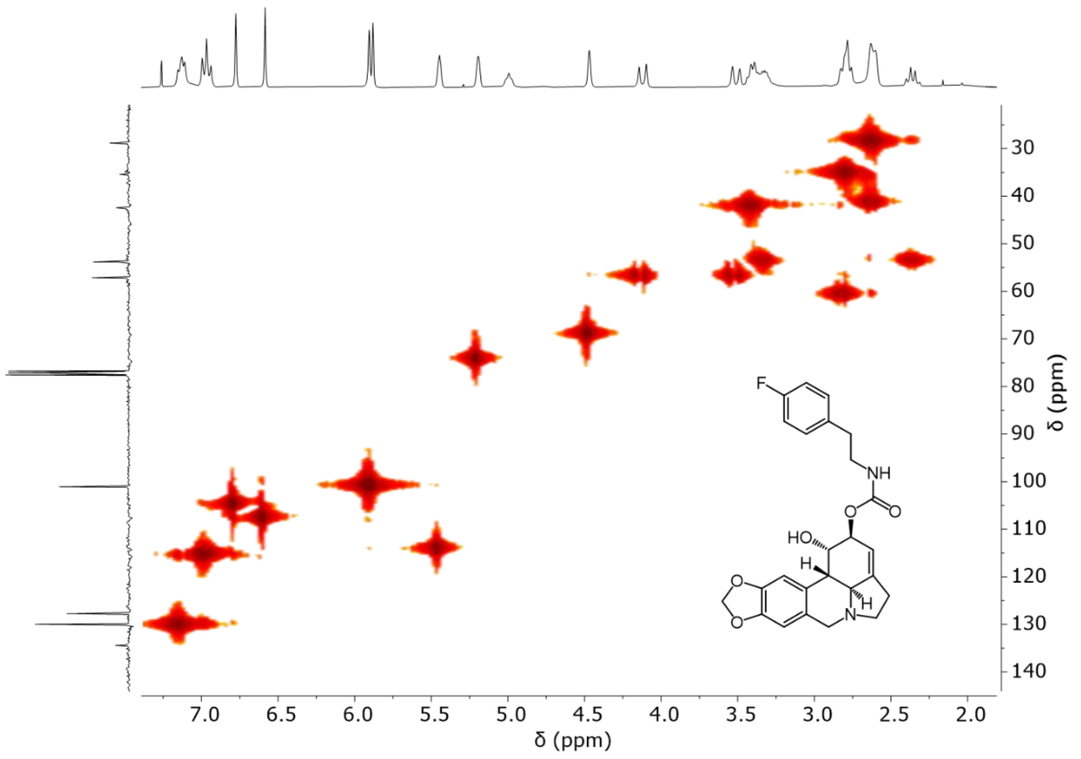

Figure S20: HMQC spectrum of compound **24**.

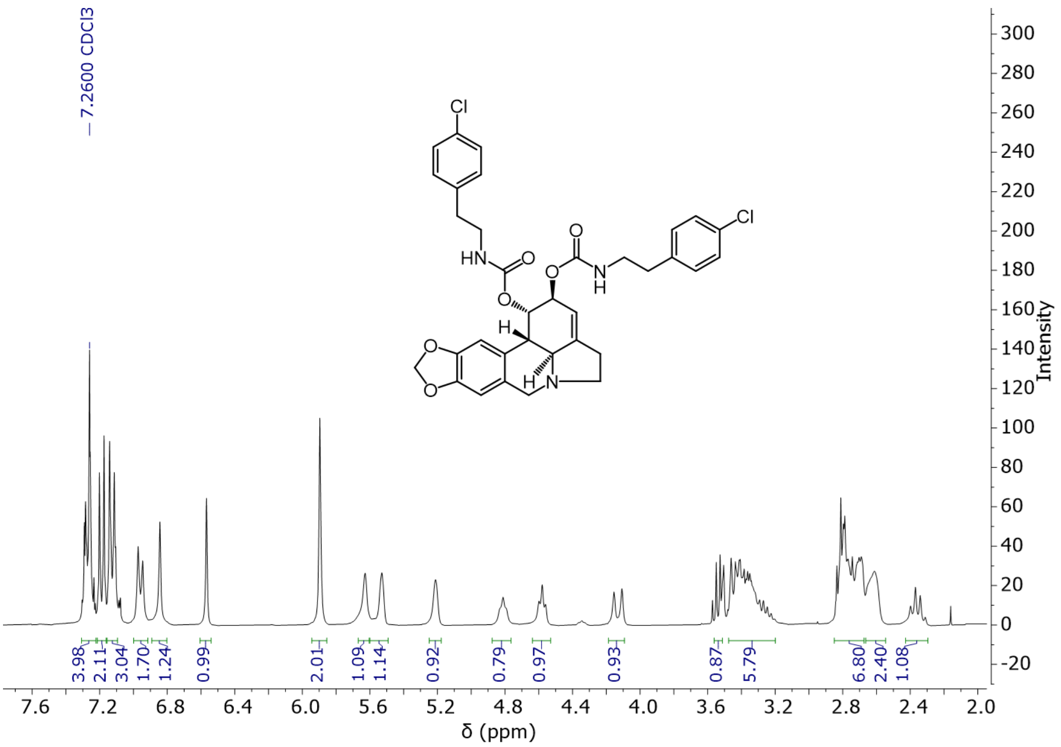

Figure S21: ^{1}H-NMR spectrum of compound **25** (300 MHz, $CDCl_3$).

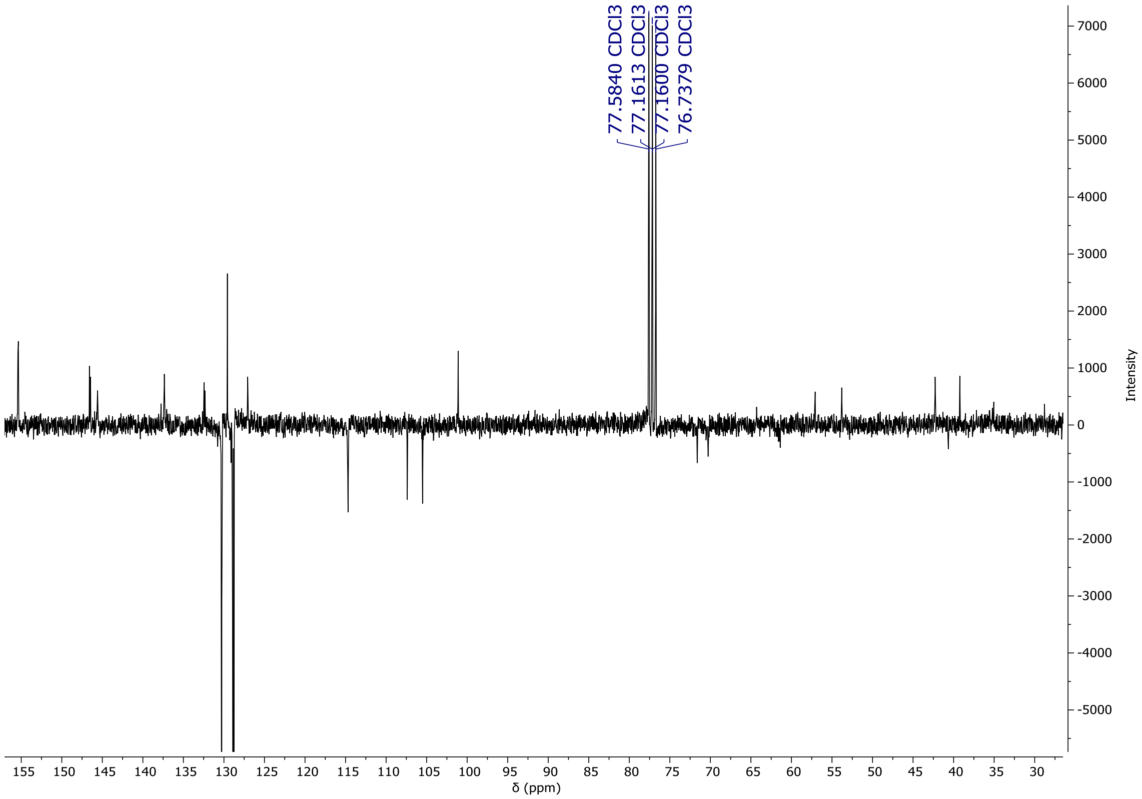

Figure S22: ^{13}C-APT NMR spectrum of compound **25** (75 MHz, $CDCl_3$).

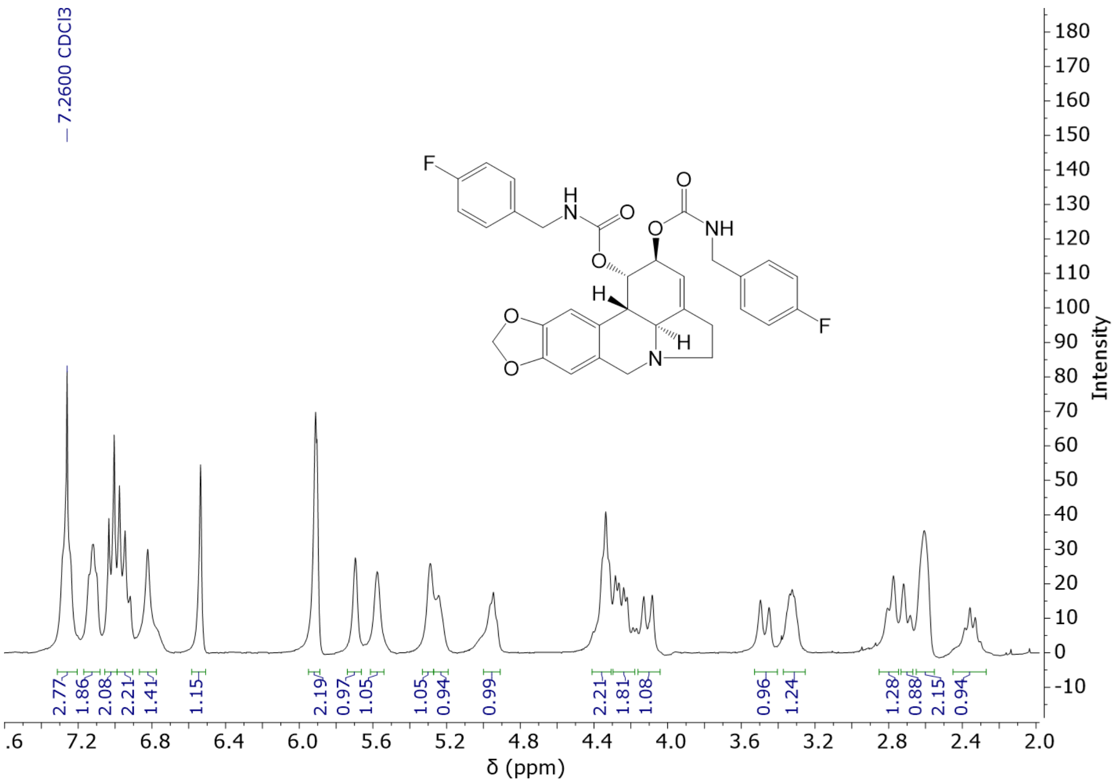

Figure S23: ^{1}H-NMR spectrum of compound **27** (300 MHz, $CDCl_3$).

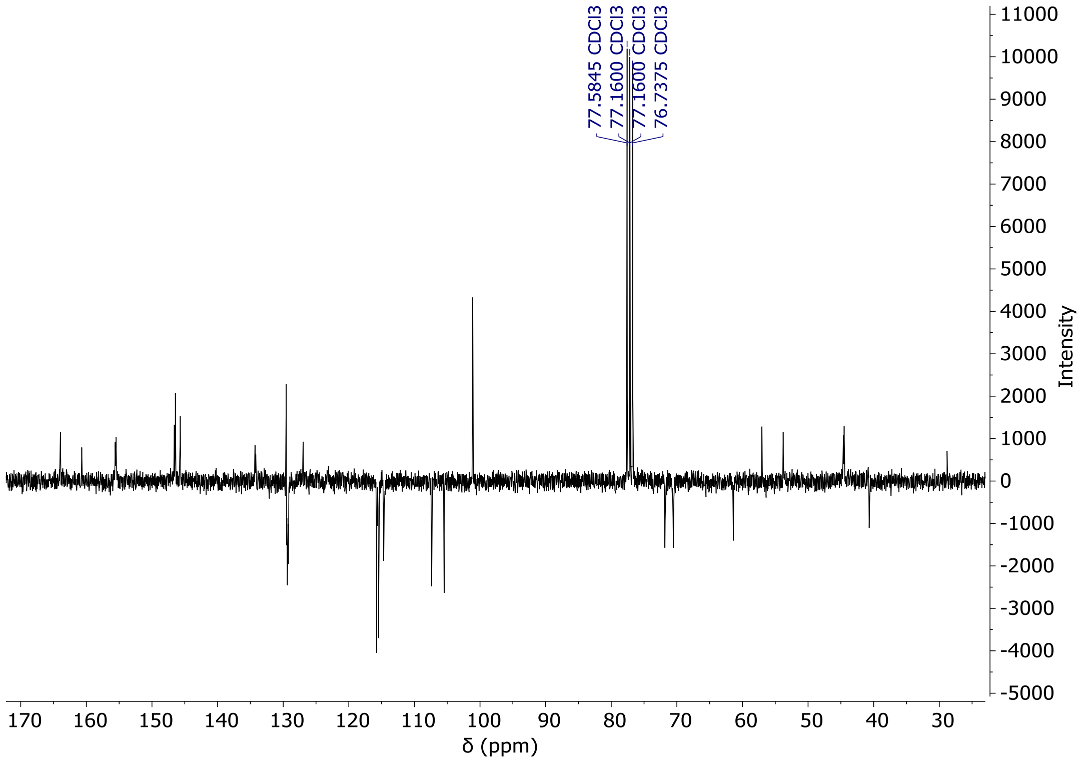

Figure S24: ^{13}C-APT NMR spectrum of compound **27** (75 MHz, $CDCl_3$).

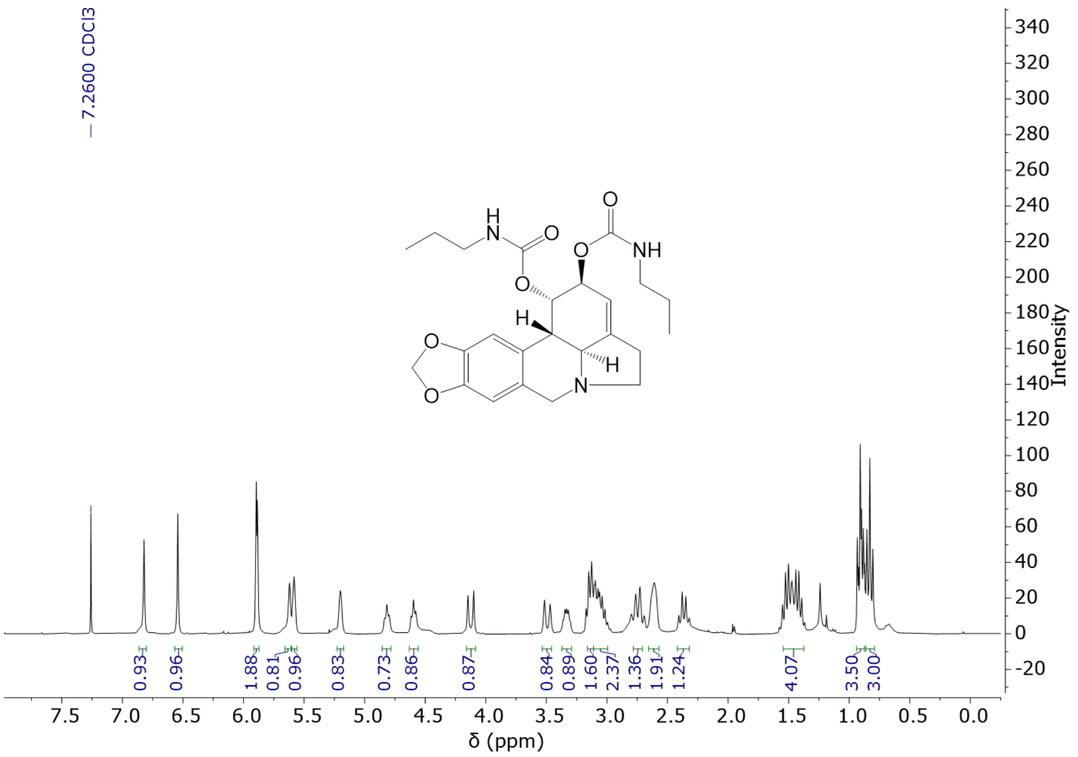

Figure S25: ^{1}H-NMR spectrum of compound **29** (300 MHz, $CDCl_3$).

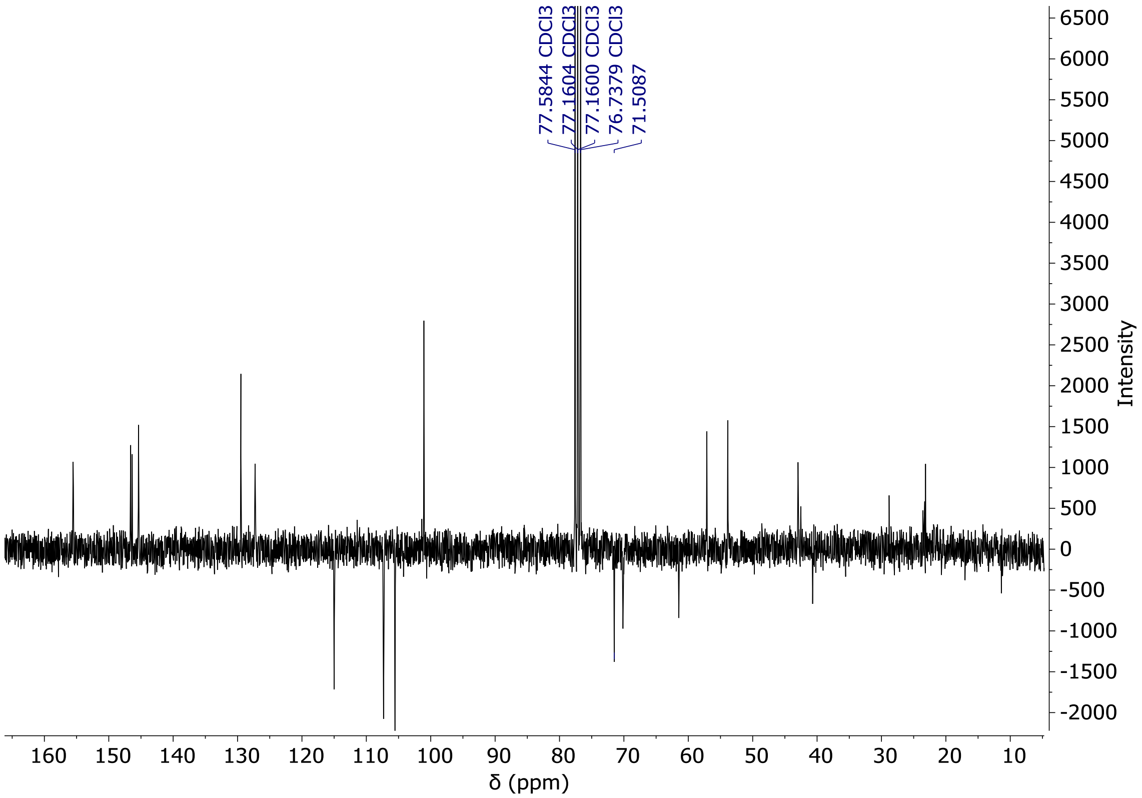

Figure S26: ^{13}C-APT NMR spectrum of compound **29** (75 MHz, $CDCl_3$).

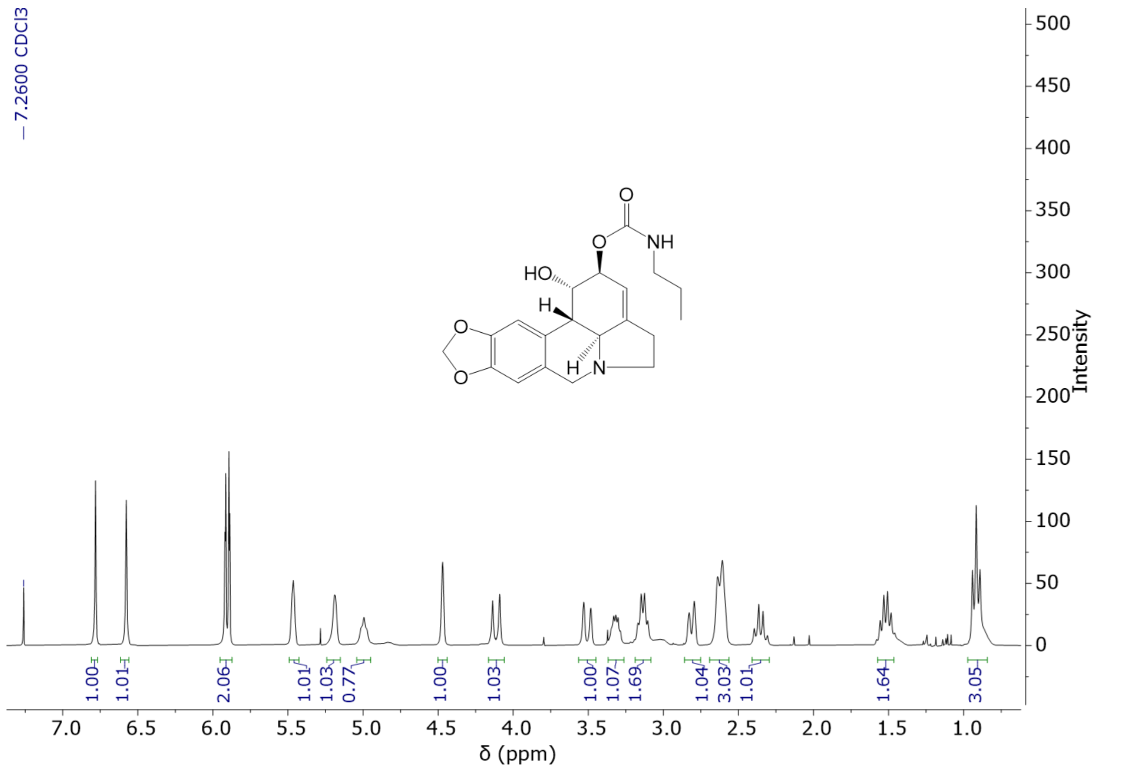

Figure S27: ^{1}H-NMR spectrum of compound **27** (300 MHz, CDCl3).

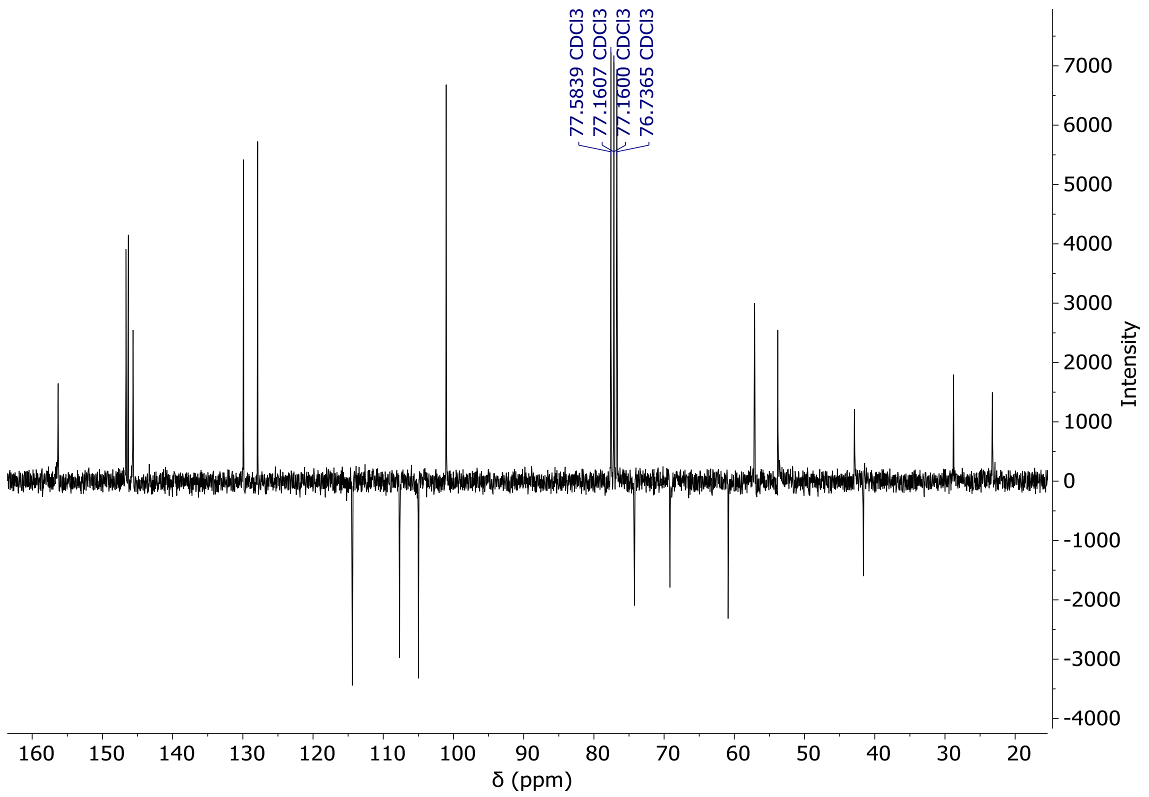

Figure S28: [13]**C**-APT NMR spectrum of compound **29** (75 MHz, CDCl3).

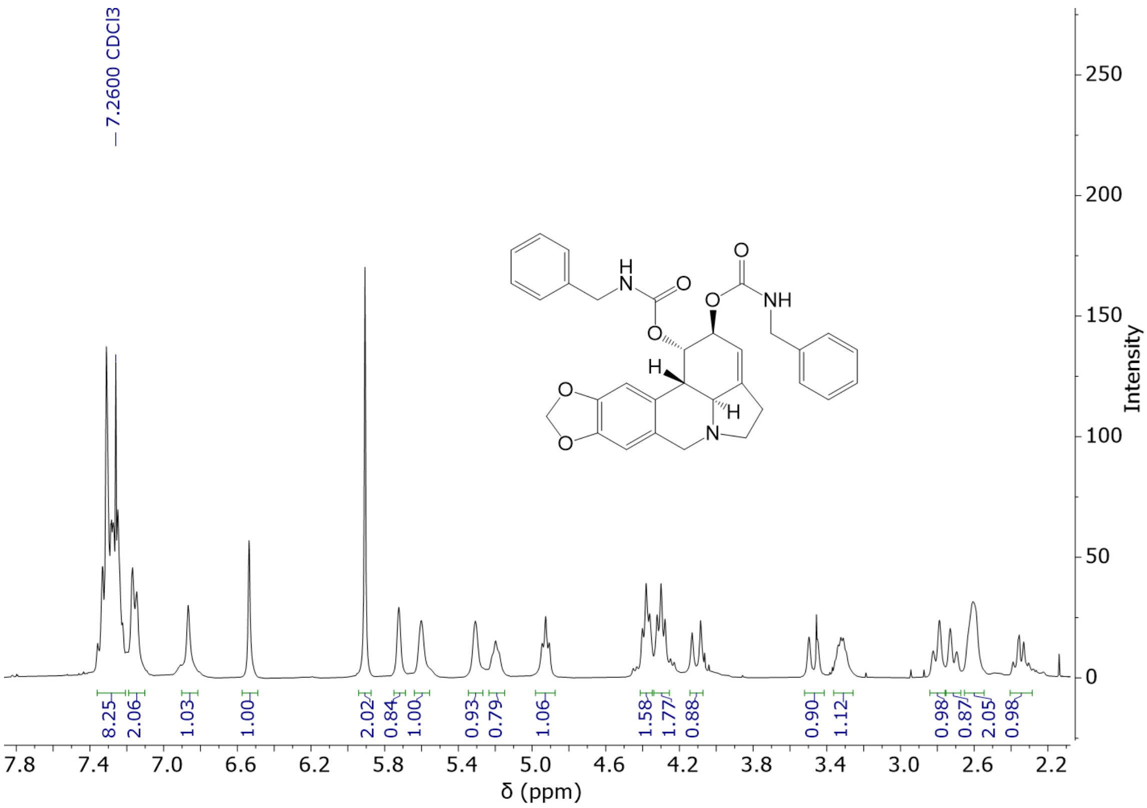

Figure S29: ^{1}H-NMR spectrum of compound **32** (300 MHz, $CDCl_3$).

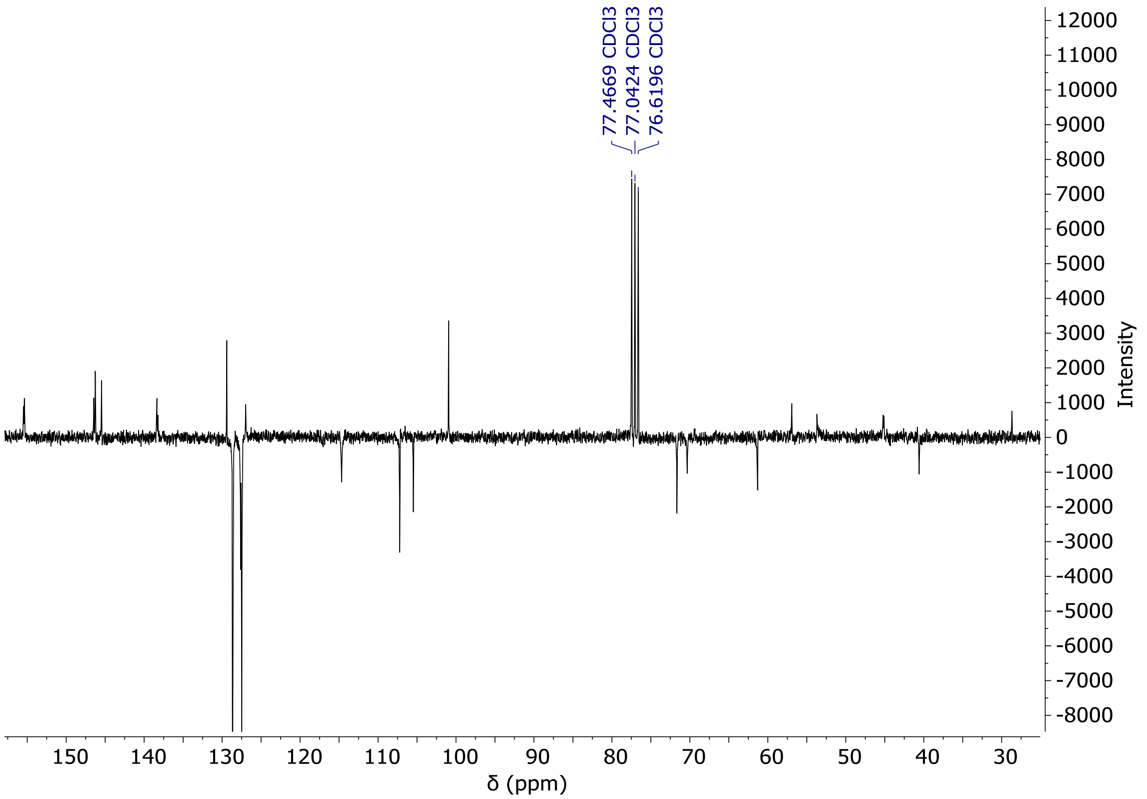

Figure S30: ^{13}C-NMR spectrum of compound **29** (75 MHz, $CDCl_3$).

3. Rhodamine-123 accumulation assay (compounds 1 – 32)

Table S1: P-pg inhibitory activity of compounds **1** – **32** on multidrug resistant human adenocarcinoma cells Colo320.

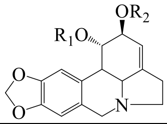

Compound	R_1	R_2	Conc (μM)	FAR[a]	FSC[b]	SSC[c]	FL-1[d]
**Colo205**	-	-	-	-	2141	758	107.0
**Colo320**	-	-	-	-	1993	1048	4.10
**1**	Lycorine		0.2	1.70	2056	1066	9.88
			2	1.74	2048	1074	7.75
			20	1.31	1991	1063	5.83
**2**	H	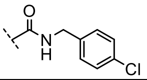	2	3.04	2000	1037	13.50
			20	7.76	1977	1041	34.40
**3**	H	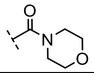	2	1.58	1995	1039	7.01
			20	1.63	1965	1038	7.26
**4**	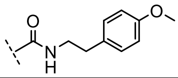	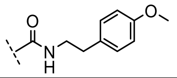	2	1.75	2009	1033	7.77
			20	3.20	1984	1064	14.20
**5**	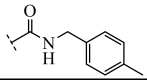	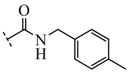	2	9.93	1733	916	45.80
			20	16.41	1707	933	77.90
**6**	H	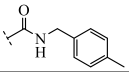	2	1.44	1745	920	7.25
			20	1.57	1742	328	11.6
**7**	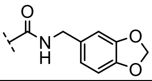	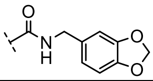	2	4.40	1767	1048	17.60
			20	10.66	1727	1082	42.60
**8**	H	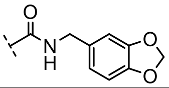	2	1.15	1732	899	5.77
			20	1.34	17.31	947	6.73
**9**	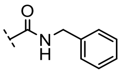	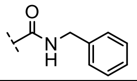	2	12.7	1933	1012	56.60
			20	17.92	1980	1053	73.60
**10**	H	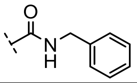	2	1.24	1750	909	6.22
			20	1.65	1807	917	12.50
**11**	H	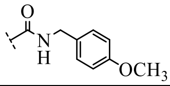	2	1.17	1760	919	5.90
			20	3.52	1748	934	17.70
**12**	H	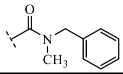	2	1.26	1755	911	6.33
			20	4.70	1723	927	23.60
**13**	H	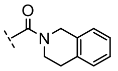	2	1.12	1758	943	5.66
			20	2.69	1716	952	13.50
**14**	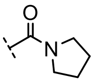	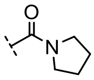	2	1.11	1743	958	5.60
			20	1.35	17.33	965	6.80
**15**	H	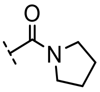	2	0.94	1747	943	4.73
			20	0.84	1738	908	4.22
**16**	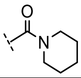	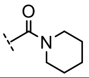	2	1.49	1979	1038	6.64
			20	3.77	1989	1070	16.70

Table S1: Continuation

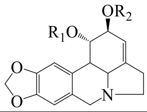

Compound	R1	R2	Conc (μM)	FAR[a]	FSC[b]	SSC[c]	FL-1[d]
**17**	H	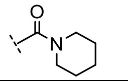	2	1.26	1759	930	6.73
			20	1.17	1711	940	5.89
**18**	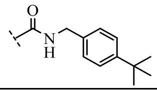	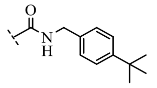	2	3.45	1783	1077	13.80
			20	8.36	17.40	1099	33.40
**19**	H	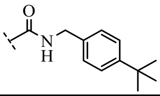	2	1.83	1950	1037	12.70
			20	7.17	2004	1081	31.80
**20**	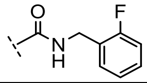	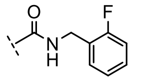	2	5.08	1773	1053	20.30
			20	13.01	1736	1012	52.00
**21**	H	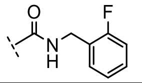	2	1.16	1788	1079	4.60
			20	2.82	1739	1040	11.30
**22**	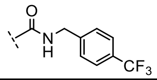	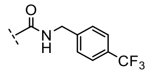	2	5.43	1760	1115	21.70
			20	13.04	1770	1119	52.10
**23**	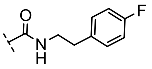	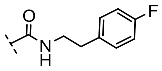	2	6.93	1766	1091	27.70
			20	14.84	1753	1106	59.30
**24**	H	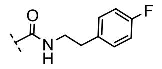	2	1.53	1787	1146	59.30
			20	4.33	1787	1146	6.15
**25**	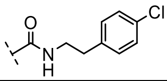	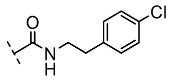	2	15.84	1980	1034	64.90
			20	21.78	1944	1101	87.00
**26**	H	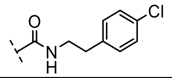	2	1.76	1992	1061	7.81
			20	6.50	2008	1085	28.80
**27**	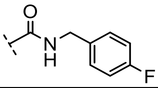	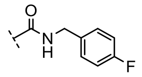	2	4.70	1787	1068	18.80
			20	11.41	1754	1056	45.60
**28**	H	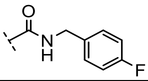	2	1.15	1783	1069	4.60
			20	1.20	1738	1049	4.82
**29**	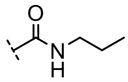	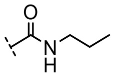	2	1.03	1782	1077	4.15
			20	0.95	1699	1005	3.82
**30**	H	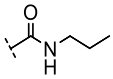	2	1.05	1780	1078	4.22
			20	0.77	1785	1114	3.09
**31**	H	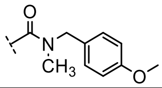	2	0.85	1815	1114	3.43
			20	2.70	1774	1097	10.80
**32**	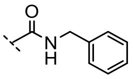	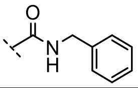	2	2.52	1773	1046	10.10
			20	9.48	1757	1066	37.90
**Verapamil**	-	-	20	7.78	1781	1074	31.10

[a]FAR (fluorescence activity ratio) values were determined by using the equation shown in section 3.4.4. Verapamil at 20μM was used as positive control. DMSO 2% (negative control) FAR = 0.89; [b]FSC: Forward scatter count of cells in the sample; [c]SSC: Side scatter count of cells in the sample; [d]FL-1: Mean fluorescence intensity of the cells.

4. Flow cytometry data

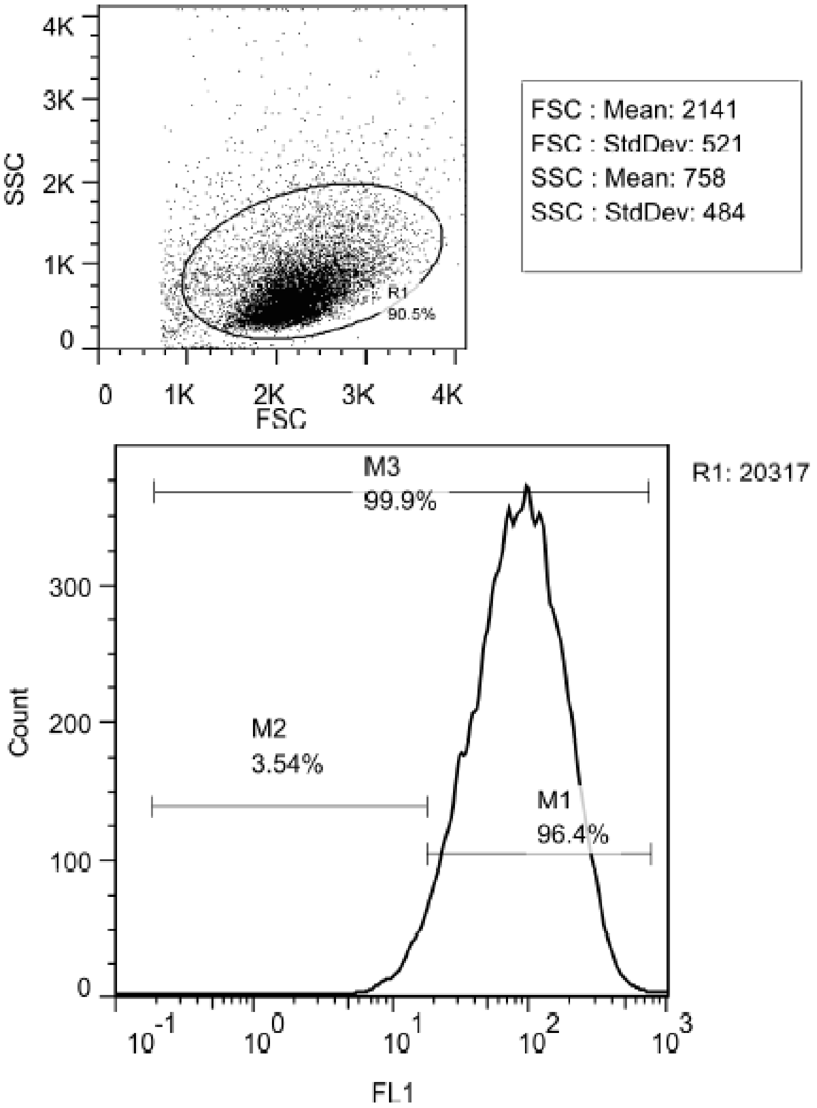

	Mean	StdDev	Count	Peak
All	104	83.0	20317	93.1
M1	107	79.5	19582	93.1
M2	13.2	3.36	719	16.0
M3	103	79.9	20300	93.1

Figure S31: Flow cytometry data for sensitive human colon adenocarcinoma cells (Colo205).

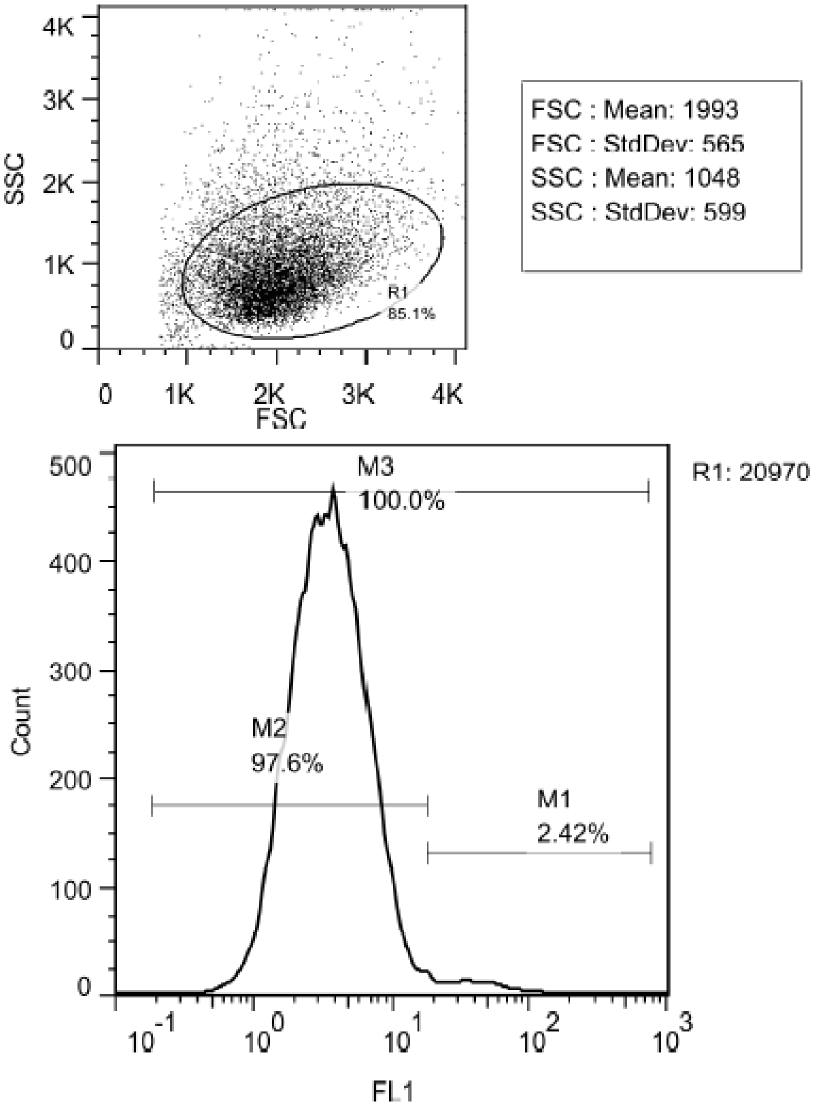

	Mean	StdDev	Count	Peak
All	5.18	9.74	20970	19.1
M1	48.5	41.5	508	19.1
M2	4.10	2.59	20459	3.79
M3	5.18	9.74	20967	3.79

Figure S32: Flow cytometry data for resistant human colon adenocarcinoma cells (Colo320).

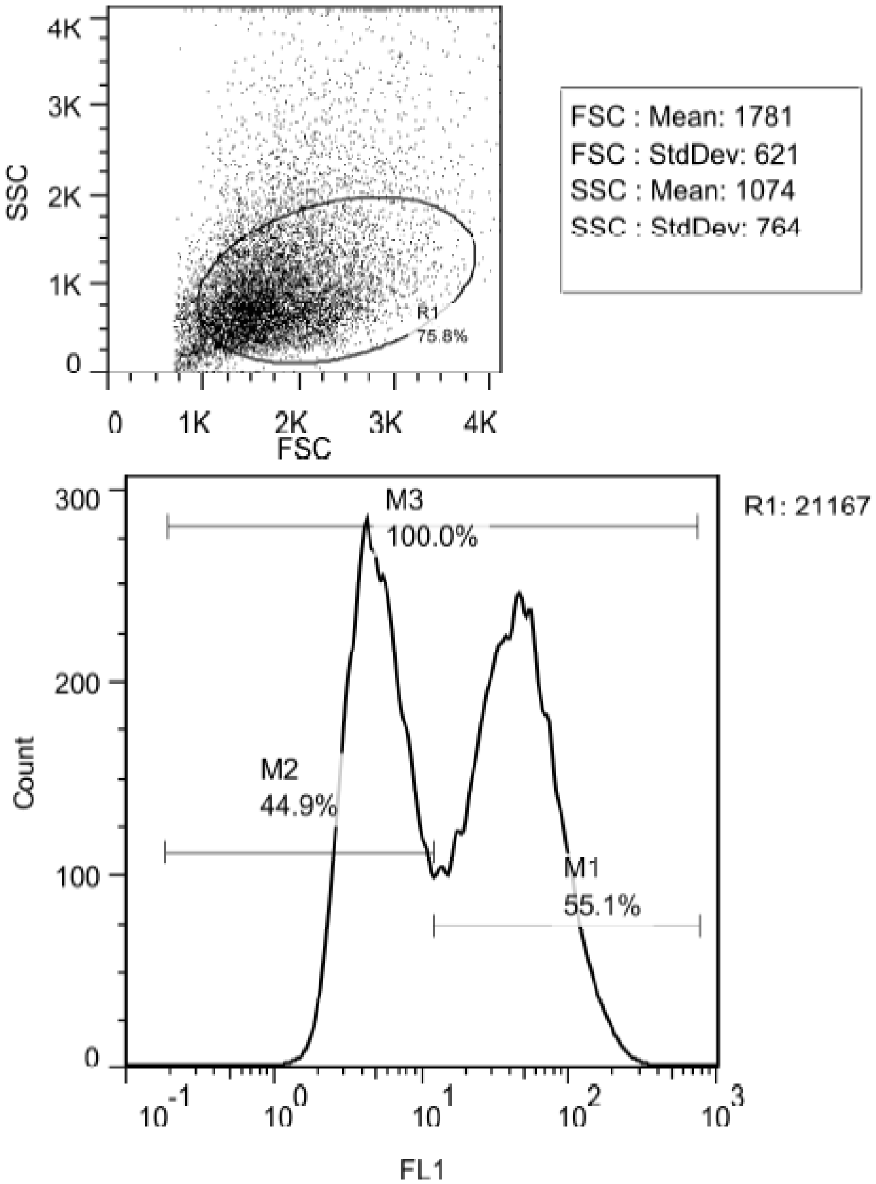

	Mean	StdDev	Count	Peak
All	31.2	38.4	21167	47.0
M1	52.0	38.3	11657	47.0
M2	5.36	2.36	9505	4.22
M3	31.1	36.7	21162	4.22

Figure S33: Flow cytometry data for verapamil (positive control) tested at 20 μM in resistant human colon adenocarcinoma cells (Colo 320).

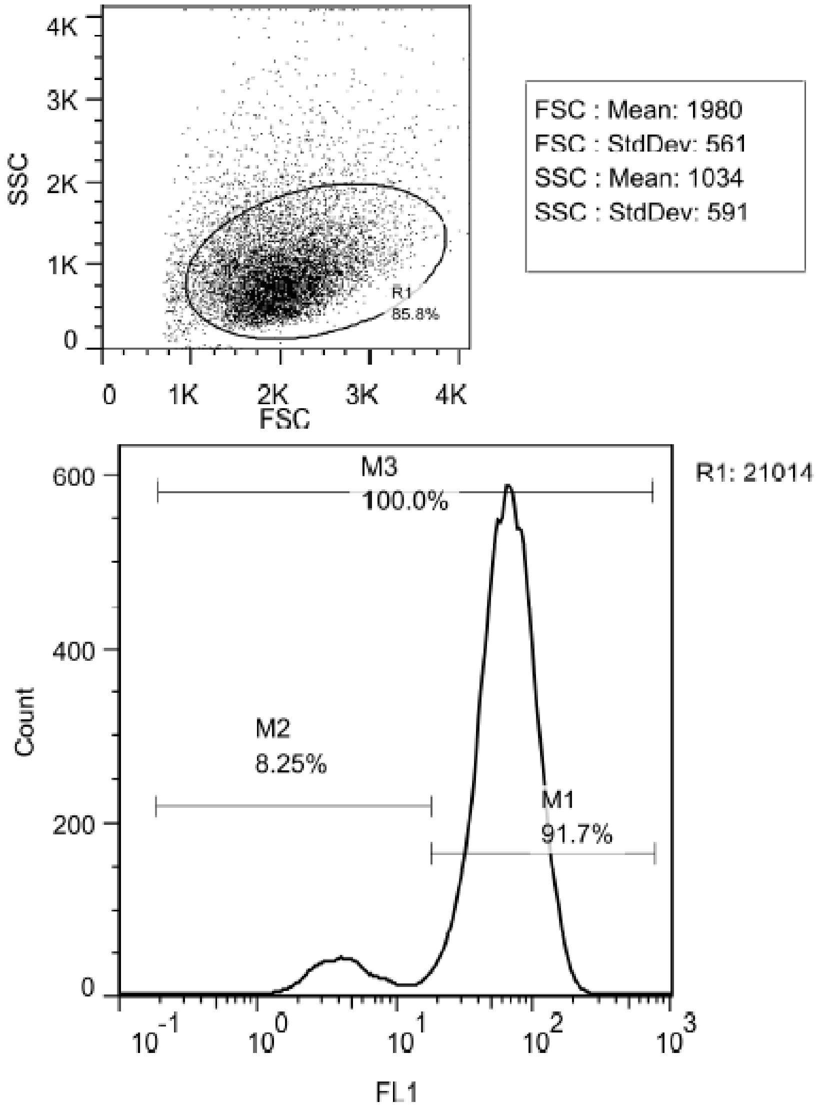

	Mean	StdDev	Count	Peak
All	64.9	35.8	21014	62.6
M1	70.2	32.4	19279	62.6
M2	5.68	3.96	1733	4.22
M3	64.9	35.8	21012	62.6

Figure S34: Flow cytometry data for compound **25** tested at 2 μM in resistant human colon adenocarcinoma cells (Colo320).

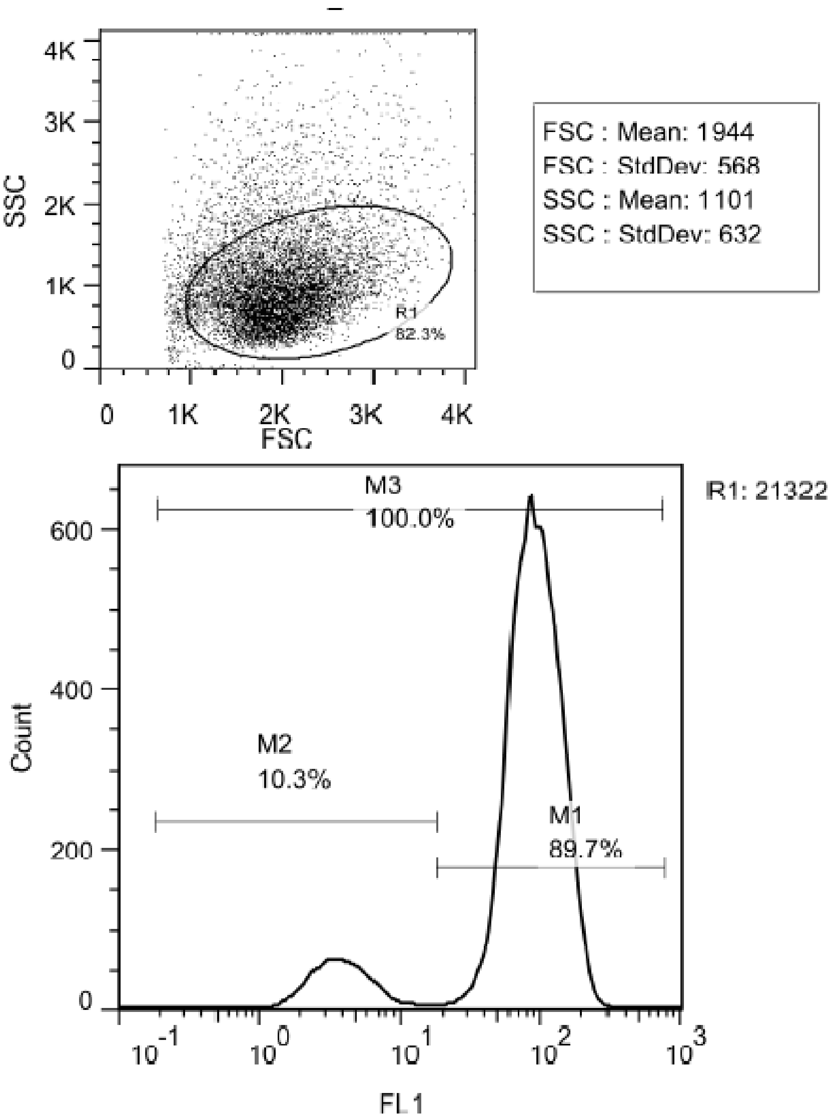

	Mean	StdDev	Count	Peak
All	87.0	46.7	21322	83.5
M1	96.5	39.4	19122	83.5
M2	4.33	2.40	2191	2.94
M3	87.0	46.7	21313	83.5

Figure S35: Flow cytometry data for compound **25** tested at 20 μM in resistant human colon adenocarcinoma cells (Colo320).

6. Combination chemotherapy results

Table S2: Effect of compounds **1**, **2**, **5**, **9**, **13**, **16**, **19**, **25**, and **26** in combination with doxorubicin on human adenocarcinoma cancer cell (Colo320).

Compound	Starting Conc. (μM)	Ratio*	CI at IC_{50}	SD	Type of interaction
**1**	2	1:1.68	1.33	0.19	Moderate antagonism
**2**	80	1:37.4	0.88	0.08	Slight synergism
**5**	65	1:60.7	0.13	0.014	Strong synergism
**9**	20	1:19	0.56	0.099	Synergism
**13**	156	1:289.6	0.69	0.089	Synergism
**16**	54	1:49.3	0.57	0.20	Synergism
**19**	15	1:28.3	0.88	0.20	Slight synergism
**25**	6	1:5.8	0.50	0.05	Synergism
**26**	76	1:35.1	0.88	0.18	Slight synergism

*Ratio: the applied combination and concentration of carbamates derivatives and doxorubicin. CI at IC_{50}: combination index (CI) at the 50 % growth inhibition dose (IC_{50}). CI < 0.1: very strong synergism; 0.1 < CI < 0.3: strong synergism; 0.3 < CI < 0.7: synergism; 0.7 < CI < 0.9: moderate to slight synergism; 0.9 < CI < 1.1: nearly additive; 1.10 < CI < 1.45: moderate antagonism; 1.45 < CI < 3.30: antagonism.

7. Physicochemical properties

Table S3: Physicochemical properties of compounds **1 – 32**.

**Compounds**	**TPSA**	**MW**	**Log *P***	**Nº H**		**MV**
				Acceptors	Donnors	
**1**	62.16	287.31	0.74	5	2	249.40
**2**	80.27	454.91	3.33	6	2	383.50
**3**	80.71	400.43	1.22	7	1	347.48
**4**	116.84	641.72	4.71	9	2	575.22
**5**	98.37	581.67	5.23	7	2	523.65
**6**	80.27	434.49	2.99	6	2	386.52
**7**	135.31	641.63	4.07	11	2	538.38
**8**	98.73	464.47	2.41	8	2	393.89
**9**	98.37	581.16	4.70	7	2	524.13
**10**	80.27	434.49	2.72	6	2	386.76
**11**	98.50	450.49	2.69	7	2	395.51
**12**	71.48	434.49	3.02	6	1	386.91
**13**	71.48	446.50	2.95	6	1	393.11
**14**	80.79	481.54	3.22	7	0	427.60
**15**	71.48	384.43	1.98	6	1	338.50
**16**	80.79	509.60	4.0	7	0	461.20
**17**	71.48	398.45	2.37	6	1	355.30
**18**	98.37	665.83	7.21	7	2	622.90
**19**	80.27	476.57	3.98	6	2	436.15
**20**	98.37	589.59	4.89	7	2	500.39
**21**	80.27	438.45	2.82	6	2	374.89
**22**	98.37	689.60	6.65	7	2	553.12
**23**	98.37	617.64	4.98	7	2	533.99
**24**	80.27	452.48	2.86	6	2	391.70
**25**	98.37	650.55	6.00	7	2	551.20
**26**	80.27	468.93	3.37	6	2	400.30
**27**	98.37	589.59	4.89	7	2	500.39
**28**	80.27	438.45	2.82	6	2	374.89
**29**	98.37	457.52	3.03	7	2	414.44
**30**	80.27	372.42	1.89	6	2	331.92
**31**	80.71	464.51	3.03	7	1	412.45
**32**	98.37	4.61	4.62	7	2	490.53

TPSA, MW, and MV were determined by using Molispiration Cheminformatics (version September 2022, https://www.molinspiration.com/cgi-bin/properties).

Log *P* was determined by using pkCSM software (version September 2022, https://biosig.lab.uq.edu.au/pkcsm/prediction) [1].

8. Pharmacokinetic properties

Table S4: Calculated pharmacokinetic properties of verapamil and compounds **2 – 32.**

Compounds	Log *S* (mol/L)	Caco-2 Permeability (log Papp in 10^{-6} cm/s)	Intestinal absorption (%)	Fractional unbound (fu)	CNS permeability (log PS)	CYP3A4 inhibitor (Yes/No)
**Verapamil**	– 5.13	1.36	94.23	0	– 2.49	Yes
**2**	– 3.79	1.09	93.28	0.12	– 2.29	No
**3**	– 2.50	– 0.19	52.26	0.422	– 3.36	No
**4**	– 4.61	0.87	91.35	0.043	– 3.23	Yes
**5**	– 4.86	0.83	98.6	0.0	−2.94	Yes
**6**	– 3.65	1.13	94.73	0.13	−2.33	No
**7**	– 3.53	1.04	97.6	0.15	−3.47	Yes
**8**	– 3.52	0.96	93.51	0.15	−3.31	Yes
**9**	– 4.58	1.01	100	0.008	−2.79	Yes
**10**	– 3.78	0.99	95.18	0.089	−2.48	Yes
**11**	– 3.67	1.055	95.62	0.151	−3.18	Yes
**12**	– 3.72	0.99	94.45	0.10	−2.32	No
**13**	– 3.39	1.05	95.02	0.12	−2.17	No
**14**	– 3.20	1.19	95.05	0.33	−3.23	No
**15**	– 2.45	1.07	95.94	0.39	−2.51	No
**16**	– 3.48	1.09	94.27	0.30	−3.08	Yes
**17**	– 2.67	1.07	95.56	0.37	−2.42	No
**18**	– 4.45	0.96	100	0.046	−2.50	Yes
**19**	– 4.40	1.07	93.16	0.026	−2.09	Yes
**20**	– 4.58	1.01	100	0.008	−3.10	Yes
**21**	– 3.71	1.03	93.8	0.106	−3.10	No
**22**	– 4.65	0.84	95.2	0.0	−2.79	Yes
**23**	– 4.92	0.94	100	0.10	−3.10	Yes
**24**	−3.90	1.01	94.6	0.13	−3.13	Yes
**25**	– 4.92	0.82	100	0.0	−2.82	Yes
**26**	−3.98	1.09	93.80	0.10	−2.37	Yes
**27**	– 4.61	0.91	100	0.10	−3.09	Yes
**28**	– 3.71	0.98	94.13	0.16	−3.11	Yes
**29**	– 3.53	0.90	94.91	0.30	– 3.27	Yes
**30**	– 2.61	1.04	95.67	0.37	−2.69	No
**31**	– 3.78	1.07	95.41	0.14	−3.12	Yes
**32**	– 4.61	1.07	100	0	−2.95	Yes

Pharmacokinetic values were obtained using pkCSM software after conversion SMILES format, as described by Pires et al. [2]

9. References

1. Pires, D.E. V.; Blundell, T.L.; Ascher, D.B. PkCSM: Predicting Small-Molecule Pharmacokinetic and Toxicity Properties Using Graph-Based Signatures. *J. Med. Chem.* **2015**, *58*, 4066–4072.
